# Analysis of QTL DM4.1 for Downy Mildew Resistance in Cucumber Reveals Multiple subQTL: A Novel *RLK* as Candidate Gene for the Most Important subQTL

**DOI:** 10.3389/fpls.2020.569876

**Published:** 2020-10-22

**Authors:** Jeroen A. Berg, Freddy W. K. Hermans, Frank Beenders, Lina Lou, Wim H. Vriezen, Richard G. F. Visser, Yuling Bai, Henk J. Schouten

**Affiliations:** ^1^Plant Breeding, Wageningen University & Research, Wageningen, Netherlands; ^2^Nunhems Netherlands BV, Nunhem, Netherlands; ^3^Jiangsu Key Laboratory for Horticultural Crop Genetic Improvement, Institute of Vegetable Crops, Jiangsu Academy of Agricultural Sciences, Nanjing, China

**Keywords:** downy mildew (*Pseudoperonospora cubensis*), cucumber (*Cucumis sativus*), plant–pathogen interactions, PI 197088, QTL mapping, transcriptomics, *receptor-like kinase* (*RLK*), *leaf rust kinase 10-like* (*LRK10L*)

## Abstract

One of the biggest problems in cucumber cultivation is cucurbit downy mildew (DM), caused by the obligate biotroph *Pseudoperonospora cubensis*. Whereas DM in cucumber was previously efficiently controlled by the *dm-1* gene from Indian cucumber accession PI 197087, this resistance was broken by new DM strains, prompting the search for novel sources of resistance. A promising source of resistance is the wild cucumber accession PI 197088. It was previously shown that DM resistance in this genotype inherits polygenically. In this paper, we put the focus on one of the QTL, DM4.1 that is located on chromosome 4. QTL DM4.1 was shown to consist of three subQTL: DM4.1.1 affected pathogen-induced necrosis, DM4.1.2 was shown to have an additive effect on sporulation, and DM4.1.3 had a recessive effect on chlorosis as well as an effect on sporulation. Near-isogenic lines (NILs) were produced by introgressing the subQTLs into a susceptible cucumber line (HS279) with good horticultural traits. Transcriptomic analysis revealed that many genes in general, and defense pathway genes in particular, were differentially expressed in NIL DM4.1.1/.2 compared to NIL DM4.1.3 and the susceptible parent HS279. This indicates that the resistance from subQTL DM4.1.1 and/or subQTL DM4.1.2 likely involves defense signaling pathways, whereas resistance due to subQTL DM4.1.3 is more likely to be independent of known defense pathways. Based on fine-mapping data, we identified the *RLK* gene *CsLRK10L2* as a likely candidate for subQTL DM4.1.2, as this gene was found to have a loss-of-function mutation in the susceptible parent HS279, and was strongly upregulated by *P. cubensis* inoculation in NIL DM4.1.1/.2. Heterologous expression of this gene triggered necrosis, providing further evidence that this gene is indeed causal for subQTL DM4.1.2.

## Introduction

The oomycete *Pseudoperonospora cubensis* [(Berk. and Curt.) Rost.] belongs to the family Peronosporaceae. Obligate biotrophs within the Peronosporaceae, such as *P. cubensis*, are commonly referred to as downy mildew (DM) pathogens (Clark and Spencer-Phillips, 2000). The host range of *P. cubensis* includes ca. 20 genera and at least 50 species within the Cucurbitaceae family, including economically important crops such as cucumber (*Cucumis sativus*), melon (*Cucumis melo*), watermelon (*Citrullus lanatus*), and squash (*Cucurbita* spp.) (Lebeda and Cohen, 2011). DM is considered to be the most important disease in cucumber worldwide, as it causes up to 100% of yield loss, and strains of *P. cubensis* have become resistant against fungicides as well as have overcome resistance in cucumber germplasm (Savory et al., 2011).

Cucumber is thought to have been domesticated ca. 3000 years ago in India, the center of origin for this species. Wild cucumber (*C. sativus* var. *hardwickii*) still occurs in northern India as well as southern China (Naegele and Wehner, 2016). During domestication, cucumber went through several genetic bottlenecks, causing a strong reduction in genetic diversity, likely due to a small initial population size, combined with a very strong selection pressure, e.g., for absence of bitterness and presence of longer fruit (Qi et al., 2013). The primary source of disease resistance is in (semi)wild cucumber accessions, maintained by gene banks, as these often carry resistance alleles that might have been lost during cucumber domestication. For over four decades, DM in cucumber was efficiently controlled by the recessive *dm-1* gene, introgressed in modern cultivars from Indian *C. sativus* var. *hardwickii* accession PI 197087 (Barnes and Epps, 1954; van Vliet and Meysing, 1974). However, due to a more virulent strain of *P. cubensis*, *dm-1* resistance provides only some level of intermediate resistance (Cohen et al., 2015; Holmes et al., 2015).

In order to identify novel sources of DM resistance, 1300 cucumber cultigens (accessions, breeding lines, as well as elite cultivars) in the USDA Agriculture Research Service collection were evaluated in multi-year, multi-location experiments. The consistently most resistant genotypes were accessions PI 197088 and PI 605996 (both of Indian origin) and PI 330628 (originating from Pakistan) (Call et al., 2012).

It was shown that in F2 populations derived from crosses among these three accessions, significant numbers of plants scored as susceptible, indicating that the resistance in these three highly resistant lines is likely conferred by different genes (Vandenlangenberg, 2015). Several groups mapped a series of QTL in accession PI 197088. The overall conclusion is that resistance in PI 197088 is polygenic, and that some QTL (notably QTL on chromosome 5 and 4) were identified by most groups as having the largest effect. However, the contribution of the different identified QTL to overall DM resistance varied greatly from study to study, possibly reflecting differences in inoculum strains in different parts of the world, and/or differences in experimental design and environmental conditions between studies (Caldwell et al., 2011; Yoshioka et al., 2014; Li et al., 2018; Wang et al., 2018). Indeed it was found that the inheritance of PI 197088-derived resistance was partially isolate-dependent (Cohen et al., 2015). DM resistance was also mapped in PI 330628, again identifying loci on chromosomes 4 and 5 as major QTL, at similar intervals compared to those of PI 197088 (Wang et al., 2016).

In our study, we focused on a QTL on chromosome 4 from PI 197088 (DM4.1), which was found by most previously published QTL mapping studies (Caldwell et al., 2011; Li et al., 2018; Wang et al., 2018) as having large or moderate effects. In order to identify the causal gene(s) for a QTL, it is advisable to reduce genetic variation due to other QTL by creating near-isogenic lines (NILs) in a uniform genetic background, which turns the quantitative effect of the QTL in a more discretely inherited Mendelian trait (Remington et al., 2002). Recently, two QTL for DM resistance on chromosomes 4 and 5 from resistant cucumber accession PI 330628 were fine-mapped to intervals containing only 13 and three predicted genes, respectively, by developing NIL-derived segregating families (Wang, 2017).

Traditionally, plant breeding has focused on dominant resistance (R) genes, conferring qualitative resistance against pathogens. Many R genes were cloned and characterized. The majority of the cloned R genes (80%) encode either intracellular proteins with nucleotide-binding and leucine rich repeat domains (NLRs) or plasma membrane-bound receptor-like kinases (RLKs) (Kourelis and Van Der Hoorn, 2018). The first group, the NLRs, trigger immune signaling by either direct recognition of cognate pathogen-encoded effector proteins, or indirect recognition of effector-mediated alterations of host proteins. NLR mediated defense signaling leads to effector-triggered immunity (ETI) which often involves the hypersensitive response (HR) leading to programmed cell death (Jones and Dangl, 2006). Interestingly, whereas most plant genomes encode hundreds of predicted *NLR* genes, the cucumber genome was found to encode only 57 predicted *NLR* genes, and similarly low numbers were found in other cucurbit species (Wan et al., 2013). Whereas some of these *NLR* genes might indeed confer resistance against pathogens, it is likely that cucumber relies on other types of genes more than other plant species for conferring resistance.

The second largest group of cloned resistance genes encode plasma membrane localized RLKs and receptor-like proteins (RLPs) (Kourelis and Van Der Hoorn, 2018). RLKs are proteins with a single transmembrane helix, a variable extracellular domain, and a rather conserved intracellular kinase domain. RLPs are essentially RLKs without a (functional) kinase domain, and were shown to be independently evolved from RLKs on multiple occasions (Shiu and Bleecker, 2003). RLKs play important roles not only in disease resistance, but also in growth and development (Tang et al., 2017). The extracellular domains of RLKs/RLPs involved in resistance recognize either apoplastic pathogen effectors or conserved microbe-associated and damage-associated molecular patterns (MAMPs and DAMPs, respectively). Traditionally, a distinction was made between ETI and PAMP-triggered immunity (PTI), in the sense that a weaker PTI response confers basal resistance against large groups of pathogens (e.g., fungal resistance by chitin perception or bacterial resistance by flagellin perception) whereas a stronger ETI response confers specific resistance against adapted pathogen species (Jones and Dangl, 2006). However, the identification of broadly conserved effectors and narrowly conserved PAMPs has shown that this dichotomy is an oversimplification (Thomma et al., 2011).

*RLK* genes form one of the most abundant gene families in plant genomes, with model organism *Arabidopsis thaliana* having over 600 predicted *RLK* genes. RLKs have diverse extracellular domains, and RLKs with similar extracellular domains are usually also more similar to one another regarding kinase domains, indicating that they form monophyletic subfamilies. Based on both the kinase-phylogeny of RLKs and their extracellular domains, 46 different RLK subfamilies were proposed (Shiu and Bleecker, 2003), although for the far majority of RLKs, both the recognized extracellular stimulus as well as the downstream targets of the kinase domain are still unknown. The most expanded and therefore well-known subfamily is that of the LRR-RLKs, which have a leucine rich repeat domain similar to that of the NLRs, allowing them to bind and recognize a wide variety of proteins and peptides. It was found that the cucumber genome encodes 178–192 LRR-RLKs as well as 42–56 LRR-RLPs, several of which are encoded by genes located within resistance QTL (Wang et al., 2014). The second-most well-known RLK subfamily is that of L-type lectin RLKs (LecRKs), the extracellular domains of which resemble soluble legume lectins which are involved in oligosaccharide binding. Several LecRKs are involved in plant defense (Bouwmeester et al., 2011; Desclos-Theveniau et al., 2012), whereas others are involved in plant growth and development, or differentially expressed upon abiotic stresses (Bouwmeester and Govers, 2009). The cucumber genome was found to encode 25 LecRKs, several of which were found to be induced by the pathogens *Phytophthora melonis* and *Phytophthora capsici* (Wu et al., 2014).

In this report, we fine-mapped a QTL for DM resistance from PI 197088, and studied a previously mis-annotated *RLK* from the *LRK10L2*-subfamily as a candidate gene for this QTL.

## Materials and Methods

### Plant Materials and Growing Conditions

Plant introduction line PI 197088, highly resistant to DM caused by *P. cubensis* (Call et al., 2012), was originally collected in Assam, India on 16 April 1951 and were obtained from the United States National Plant Germplasm System (NPGS). Homozygous breeding line HS279 is a pickling type cucumber, susceptible to DM, with good horticultural characteristics, and was supplied by Nunhems Netherlands BV.

A marker-assisted backcrossing strategy was employed in order to generate NILs and segregating populations. In an F3 population derived from a cross between PI 197088 (male parent) and HS279 (female parent), a partially resistant individual with a recombination event close to QTL DM4.1 was selected. This F3 individual was backcrossed to recurrent parent HS273 for three generations, using marker assisted selection with SNP markers for background selection of HS279 alleles and foreground selection of PI197088 alleles in the DM4.1 interval. A resulting F3BC3 individual fixed for HS279 alleles at all markers except for the DM4.1 introgression was self-fertilized for two generations to generate F3BC3S2 populations, which were genotyped with SNP markers within the DM4.1 interval in order to select 19 individuals with recombination events. Recombinant F3BC3S2 individuals were self-fertilized in order to generate segregating F3BC3S3 families, which were used as fine-mapping populations. Several informative heterozygous and homozygous F3BC3S3 individuals were selected to generate segregating RHLs and fixed NILs, respectively.

Unless otherwise indicated plants were grown on blocks of rockwool in climate chambers with temperatures of 22°C (day) and 17°C (night), with a 16/8 h day/night cycle, and a relative humidity of 80%.

### *P. cubensis* Inoculum Maintenance, Disease Tests, and Phenotyping

An isolate of *P. cubensis* obtained from an infected cucumber field in Haelen, Netherlands, in 2018, was maintained on fully expanded cucumber leaves, healthy in appearance before inoculation. For pathogen maintenance, detached leaves were kept in closed boxes containing water-soaked paper towels, and inoculated with a spore suspension developed as described below. Boxes containing inoculated cucumber leaves were kept in a climate chamber under 18°C (day) and 15°C (night), with a 16/8 h day/night cycle for 10 days. Heavily infected detached leaves were preserved at −20°C as inoculum source for <6 months. Spore suspensions were produced by washing spores from frozen infected leaves using tap water, and filtering through cheesecloth. The spore concentration was measured using a hemocytometer, and adjusted to 1^∗^10^4^ spores/ml.

Cucumber plants for *P. cubensis* disease tests were grown on rockwool blocks in trays containing an excess of water, in plastic tents which were closed the day before inoculation, to ensure a high relative humidity. Both sides of cucumber leaves were sprayed with spore suspension prepared as described above. After inoculation, plants were left in darkness at 18/15°C (day/night) for 24 h in closed plastic tents. At 1 dpi, plastic tents were opened, and normal climatic conditions were resumed. Starting from 7 days post inoculation, yellowing (chlorosis), sporulation, and collapsing (necrosis) of leaves were assessed by eye on a 1–9 scale, 9 being fully resistant and 1 being fully susceptible.

### QTL Analysis and Statistical Analysis Disease Scores

For QTL mapping, phenotypic data were collected on 27 F_3_BC_3_S_3_ families, of which 19 were segregating and eight were uniformly homozygous. For homozygous families, average phenotypic scores of 20 individual plants were used. For segregating families, individual scores of 20–91 plants were used. All plants were genotyped using seven SNP markers within the DM4.1 interval as well as two SNP markers flanking the interval (Supplementary Table 1). QTL were mapped for each of three phenotypes, i.e., chlorosis, sporulation, and necrosis, using the “scanone” procedure from the R/qtl package (Broman et al., 2003), and including family identifiers as a covariable to correct for the population structure.

For analysis of DM resistance in segregating RHLs, 94 plants per family were sown, of which based on SNP genotyping eight plants each were selected homozygous for the introgression, heterozygous, and homozygous for absence of the introgression. Plants were phenotyped; average scores and standard deviations were determined for the three symptoms (chlorosis, sporulation, and necrosis). Statistical analysis of phenotypic data was performed using SPSS v23 software (IBM), with non-parametric Kruskal–Wallis tests and stepwise step-down *post hoc* analysis.

### RNA Extraction, Sequencing, and Differential Expression Analysis

For RNA extractions, plants of genotypes HS279, NIL DM4.1.1/.2, and NIL DM4.1.3 were inoculated with *P. cubensis* as described above, or a mock treatment consisting of spraying leaves with tap water. Three days post inoculation, leaves of three biological replicates per genotype x treatment combination were sampled and immediately frozen in liquid N_2_. Leaf samples were stored at -80°C. Leaf samples were ground in liquid N_2_, and total RNA was isolated by using the RNeasy Kit (Qiagen, Germany). Possible DNA contamination of RNA samples was removed by treatment with DNase I, Amp Grade (Invitrogen life technologies, United States). RNA samples were shipped on dry-ice to BGI Tech Solutions (Denmark) for RNA sequencing using a BGISEQ-500 platform, resulting in ca. 50 million fastq reads (100 bp PE) per sample. Fastq reads were aligned to the cucumber reference genome (Chinese Long 9930 v2) using TopHat (v2.1.1) (Kim et al., 2013). Uniquely aligned read counts per gene per sample were determined using HTSeq-count (Anders et al., 2015). Differential expression analysis was performed in R using package DEseq2 (v3.8) (Love et al., 2014). Principal component analysis (PCA) was performed on regularized log values of read counts. As suggested in the DESeq2 vignette, treatment and genotype were combined as a single factor for the analysis of contrasts between genotypes and treatments. Differentially expressed genes were called significant using an adjusted *P*-value (Benjamini–Hochberg adjustment) and a false discovery rate of <0.05. As a threshold for biological significance, a twofold change in expression was used. Lists of up- and downregulated genes from the contrasts of the conditions were compared and used to create the Venn diagram with the overLapper function from R package systemPipeR (Backman and Girke, 2016).

SNPs in coding regions were called using Samtools mpileup (v1.3.1) (Li et al., 2009a), and the effect on coding sequences of predicted genes was annotated using SnpEff (Cingolani et al., 2012).

### Identification of Cucumber Orthologs of Defense Pathway Genes

Protein sequences of *A. thaliana* defense pathway genes *PR1* (AT2G14610), *PR2* (AT3G57260), *PR3* (AT3G12500), *PAD4* (AT3G52430), *EDS1* (AT3G48090), *NPR1* (AT1G64280), *RST1* (AT3G27670), and *PGIP2* (AT5G06870) were retrieved from The Arabidopsis Information Resource (TAIR) on www.arabidopsis.org [accessed on 13-03-2019]. Protein sequences were used as queries for BLASTp searches against reference genomes of cucumber (Chinese Long 9930 v2) and *Arabidopsis* (TAIR10) in order to identify homologous genes in both species. Multiple sequence alignments and neighbor-joining phylogenetic trees were constructed using CLC Genomics Workbench v11, with standard settings. Putative cucumber defense pathway genes were selected based on orthology with the *Arabidopsis* gene. In cases where multiple putative cucumber orthologs of an *Arabidopsis* defense pathway gene were identified, capital letters (A–D) were added to the gene names in order to discriminate between orthologs. Supplementary Table 11 gives an overview of identified putative cucumber defense pathway genes.

### Identification and Sequencing of *RLK* Structural Variation

For genomic inspection of the *RLK* region, DNA was isolated from a leaf sample obtained from genotype NIL DM4.1, using a CTAB protocol (Healey et al., 2014). DNA was shipped on dry-ice to Novogene Bioinformatics Technology Co. (Hong Kong) for Illumina HiSeqX resequencing. Obtained clean reads (100 bp PE) were aligned to the reference genome (Chinese Long 9930 v2) using Bowtie2 (v2.2.6) (Langmead and Salzberg, 2012). Reads mapping to the *RLK* locus were manually inspected using IGV (v2.3.32) (Robinson et al., 2011).

For PCR amplification of the suspected structural variation in gene Csa4M410860, DNA was isolated from three independent individuals of genotypes NIL DM4.1.1/.2 and HS279 as described above. PCR reactions were performed using DreamTaq DNA polymerase (Thermo Fisher Scientific) according to manufacturer’s protocol, with primers F 5′-TTCCCCGCGGACATCTCTA-3′ and R 5′-AGGTCAACTTTCACACAGTCCA-3′. PCR products were sent for Sanger sequencing (GATC Biotech, Germany) using the same primers.

### *In silico* Analysis of Presence 551 bp Insertion Allele *CsLRK10L-2* in Resequenced Cucumber Germplasm

Resequencing data of 115 cucumber accessions (Qi et al., 2013) were downloaded from the NCBI short read archive, accession SRA056480. For each accession, reads were aligned to a fasta file containing the genomic sequence of the *CsLRK10* locus, including the 551 bp insertion identified in NIL DM4.1.1/.2. Presence or absence of the 551 bp insertion, compared to the reference genome, was manually scored by inspection of the alignment using IGV (v2.3.32) (Robinson et al., 2011). Relevant information of lines containing the 551 bp insertion allele was obtained from the supplementary data files of Qi et al. (2013).

### Cloning and Transient Overexpression of *CsLRK10L* Genes

RNA was isolated from genotypes NIL DM4.1.1/.2 and HS279, as described above. cDNA was synthesized using Superscript III reverse transcriptase (Thermo Fisher Scientific) with oligo-dT primer, following the manufacturer’s protocol. *CsLRK10L1* and *CsLRK10L2* were amplified from both cDNA samples using Phusion high-fidelity polymerase (Thermo Fisher Scientific) according to manufacturer’s protocol with primers F 5′-CACCATGGATTCCCCAATTTCCTC-3′ (both genes) and R 5′-GGAGCTGTCTGCTATTGATGG-3′ (*CsLRK10L1*) or R 5′-AACCACAACAATCCTTAACAACC-3′ (*CsLRK10L2*). PCR products were run on agarose gels and subsequently purified from gel using the QIAquick Gel Extraction Kit (Qiagen, Germany).

Purified products were cloned into Gateway-compatible vector pENTR D-TOPO (Thermo Fisher Scientific) and transformed to chemically competent *Escherichia coli* strain One Shot TOP10. Presence of the right fragment was assessed by colony PCR using primers. Plasmids were recovered using the Qiaprep spin miniprep kit (Qiagen, Germany). Sequencing reactions were performed in duplo using pUC/M13 forward and reverse sequencing primers (GATC Biotech, Germany).

Entry plasmids were transferred using LR clonase II (Thermo Fisher Scientific) into binary vector pK7WG2, which harbors the constitutively active 35S Cauliflower Mosaic Virus promotor and the *nptII* marker gene for kanamycin resistance (Karimi et al., 2002). Recombinant plasmids were transformed to chemically competent *E. coli* strain dh5α. Positive recombinant bacterial colonies were screened by colony PCR using *CsLRK10L* specific primers as described above, and sequenced. Recombinant plasmids were recovered using the Qiaprep spin miniprep kit (Qiagen, Germany). Binary vectors were transformed to electrocompetent cells of *Agrobacterium tumefaciens* strain AGL1-virG by electroporation.

*Nicotiana benthamiana* plants were grown in a greenhouse under standardized conditions for 5 weeks. *A. tumefaciens* strains harboring the binary vectors were cultured in LB medium with appropriate antibiotics for 18 h at 28°C. Cells were collected by centrifugation, resuspended in agroinfiltration medium and adjusted to the desired concentration. To increase expression efficiency, *A. tumefaciens* strains were mixed in a 1 : 1 ratio with a strain expressing silencing suppressor *P19* (Voinnet et al., 2003). *A. tumefaciens* cultures were infiltrated in *N. benthamiana* leaves with a needleless syringe.

### Phylogenetic Analysis

Partial protein sequences of CsLRK10L1 and CsLRK10L2 predicted oligogalacturonan-binding, WAK-associated and protein kinase domains were used as BLASTp queries against translated genomes of cucumber (Chinese Long 9930 v2) and *Arabidopsis* (TAIR 10) in order to select homologous genes. Regarding kinase domain homologs of both proteins, multiple sequence alignments were performed, and maximum-likelihood phylogenetic trees were constructed using CLC Genomics Workbench v11.

Predicted proteomes of cucumber (Chinese Long 9930 v2) and *Arabidopsis* (TAIR 10) were scanned using InterProScan v5.27 against the Pfam database for presence of oligogalacturonan-binding (IPR025287) and/or WAK-associated (IPR032872) domains. Hits were extracted from the proteomes using the –getfasta option from Bedtools v2.27.1 (Quinlan and Hall, 2010), and used to construct multiple sequence alignments and maximum-likelihood phylogenetic trees using CLC Genomics Workbench v11.

## Results

### Distinguishing Different Disease Symptoms Revealed Three subQTL Within the DM4.1 Interval

**FIGURE 1:**
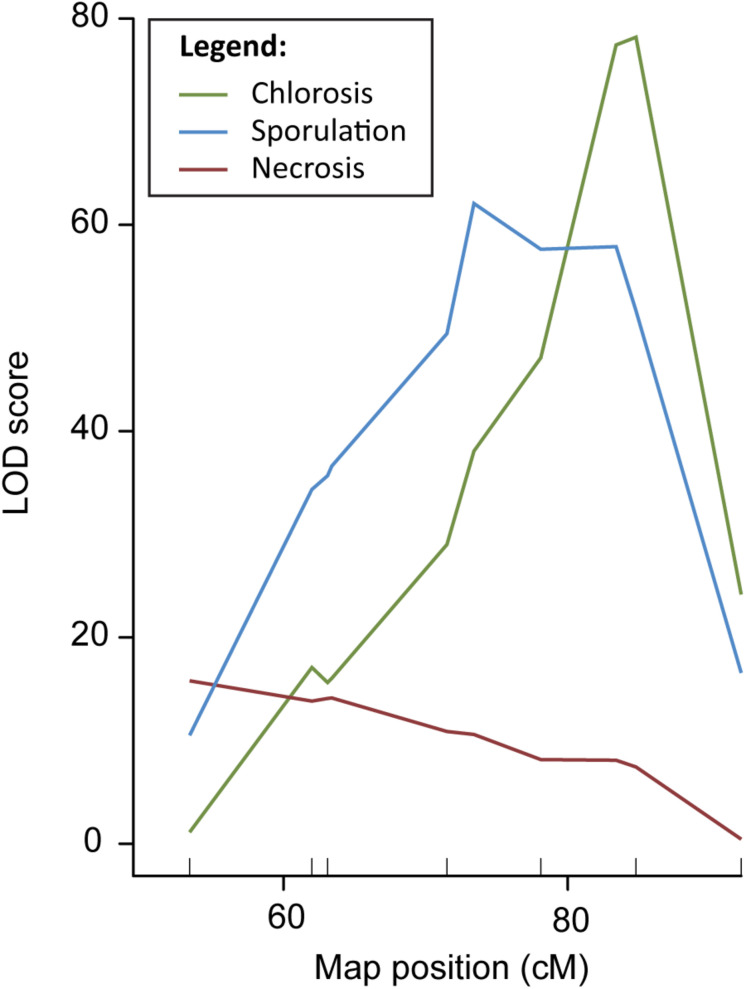
QTL analysis DM resistance within the DM4.1 interval based on multiple disease phenotypes. QTL analysis was carried out in a population consisting of 19 recombinant families derived from a QTL isogenic introgression line. The mapping population was scored for chlorosis, sporulation, and pathogen-induced necrosis, and genotypes with seven SNP markers in the DM4.1 interval. Two SNP markers flanking this interval were not polymorphic, as the progenitor of the mapping population was fixed for these alleles.

In order to fine-map QTL DM4.1, a QTL isogenic introgression line was selected originating from a cross between the DM resistant cucumber accession PI 197088 and the susceptible line HS279, followed by three generations of backcrossing with HS279 as the recurrent parent. This plant was selected because it had a (heterozygous) 12 Mb introgression on chromosome 4 corresponding to QTL DM4.1, in a homozygous, uniform HS279 background (Supplementary Table 1, marker locations). After two generations of selfing, 19 plants with recombinations between markers at Chr4:11.479.953 and Chr4:20.438.834 (based on the cucumber reference genome, Chinese Long 9930 v2; Huang et al., 2009; Li et al., 2011) were selected to develop families. Recombinant families were inoculated with *P. cubensis* in a controlled climate chamber experiment. Phenotypic data were collected on the DM inoculation response of these 19 families using three criteria: chlorosis, sporulation, and necrosis, each on a 1–9 scale with 1 being highly susceptible and 9 being highly resistant (Supplementary Figures 1, 2). Individuals were genotyped using nine SNP markers (Supplementary Table 1 and Supplementary Figure 2), seven within the QTL interval and two flanking the interval. We conducted QTL analysis with R/qtl using the “scanone” procedure for all three phenotypes individually, correcting for the presence of sub-populations by a population co-factor (Figure 1).

Although the three QTL for the different symptoms overlapped, we found that the peak positions were markedly different, indicating the potential existence of multiple causal genes, each with a different effect on the disease phenotype. Supplementary Table 1 gives physical locations of peak- and flanking markers for the QTL detected for each of the three phenotypes, which we will refer to as QTL DM4.1.1, DM4.1.2, and DM4.1.3 hereafter. For the necrosis phenotype, an interval of 2.9 Mb was determined (subQTL DM4.1.1), with the peak position at Chr4:12127169. For the sporulation trait, two peaks were detected, one major peak (subQTL DM4.1.2) at a 2.7 Mb interval with the peak position at Chr4:15766975 and a secondary peak (subQTL DM4.1.3) at a 3.6 Mb interval with the peak position at Chr4:18469868. Lastly, for the chlorosis trait, one peak was detected, at the same interval as the secondary peak for sporulation (DM4.1.3).

### Disease Tests on Segregating Populations Confirm the Presence of subQTL DM4.1.2 and DM4.1.3

**FIGURE 2:**
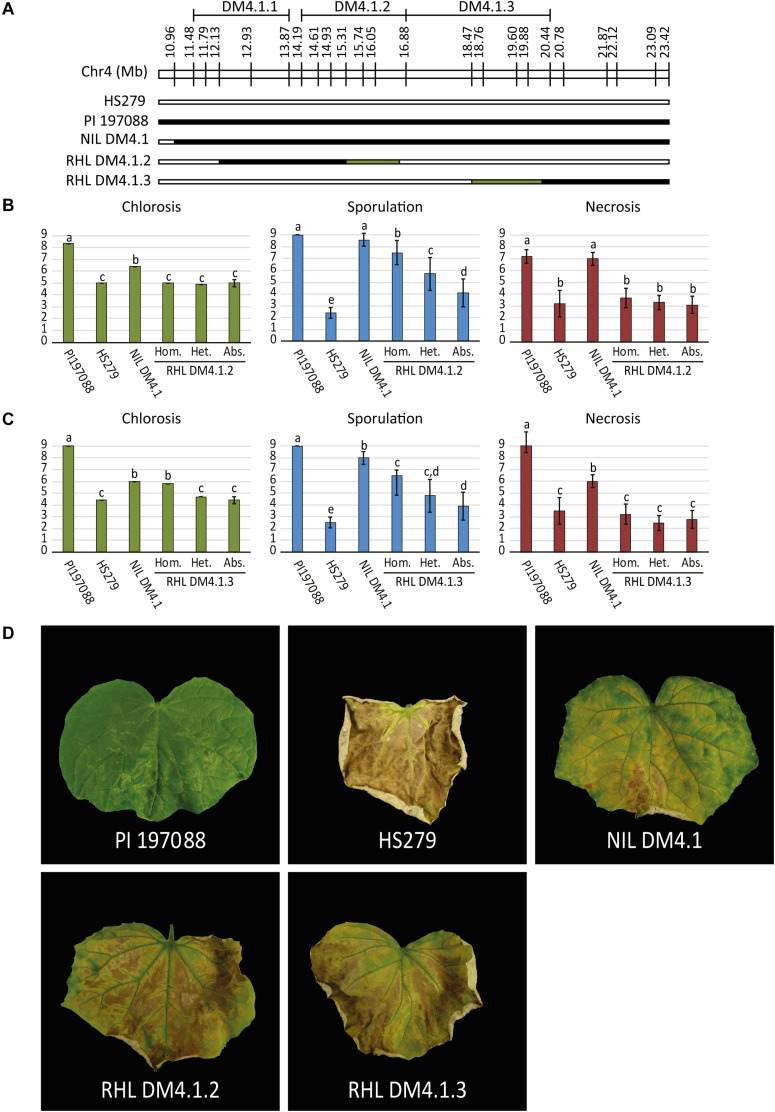
*P. cubensis* disease test on RHLs segregating for subQTL DM4.1.2 and DM4.1.3. **(A)** Residual heterozygous lines (RHLs) were developed from individuals in the mapping population heterozygous for partial introgressions individuals homozygous for the full introgression. Additionally, a homozygous individual was selected to develop a near isogenic line (NIL DM4.1). Bars represent the allele of genotypes at marker locations on the DM4.1 interval. Black bars indicate the PI 197088 allele, white bars indicate the HS279 allele, and green bars represent heterozygosity in the individuals and thus segregation in the RHLs. RHLs segregating for subQTL DM4.1.2 **(B)** or DM4.1.3 **(C)** were inoculated with *P. cubensis* and subsequently scored for chlorosis (7 dpi), sporulation (12 dpi), and necrosis (12 dpi). Eight individuals per genotype were scored. Bars represent average phenotype scores on a 1–9 scale ranging from susceptible to resistant. Error bars represent standard deviation. Bars with different letters are statistically significant from one another (Kruskal–Wallis, *p* < 0.05). **(D)** Representative photographs of disease phenotypes at 12 dpi are shown for the resistant donor PI 197088, the susceptible recurrent parent HS279, NIL DM4.1, and homozygous individuals of RHL DM4.1.2 and RHL DM4.1.3.

As the QTL data indicated the potential existence of multiple subQTL within the greater DM4.1 locus (Figure 1), we selected two heterozygous individuals from recombinant families segregating for subQTL DM4.1.2 and DM4.1.3, respectively (Figure 2A). Both individuals were selfed in order to develop segregating residual heterozygous lines (RHLs). It should be noted that the RHL segregating for presence of subQTL DM4.1.2 was fixed for the presence of an (homozygous) introgression corresponding to subQTL DM4.1.1. Additionally, one individual homozygous for the full DM4.1 introgression was selfed in order to develop a NIL (NIL DM4.1). Each of the two RHLs was inoculated with *P. cubensis* in a controlled climate chamber experiment, using resistant donor PI 197088, partial resistant NIL DM4.1, and susceptible recurrent parent HS279 as controls. Phenotypic data were collected seven (chlorosis) and 12 (sporulation and necrosis) days post inoculation on a 1–9 scale (Figures 2B,C). Individuals were genotyped using SNP markers.

Resistant donor PI 197088 was indeed very resistant to DM, showing no expanding lesions, hardly any chlorosis, and no sporulation. At later time-points, necrotic micro lesions were sometimes visible. Susceptible recurrent parent HS279 was, as expected, consistently the most susceptible genotype, with a high degree of chlorosis, abundant sporulation, and fast expanding necrotic lesions. NIL DM4.1 showed a partial resistant phenotype, with delayed chlorosis and necrosis compared to the susceptible parent, and sparse sporulation. Both RHLs with partial DM4.1 introgressions were less resistant than NIL DM4.1 with the full introgression, but more resistant than the susceptible recurrent parent (Figure 2), confirming the existence of separate subQTL underlying QTL DM4.1.

Significances of differences in disease phenotypes for both of the populations were determined using Kruskal–Wallis tests (*p* < 0.05). Step-down *post hoc* analysis revealed that in RHL DM4.1.2, significant differences (*p* < 0.05) were found regarding sporulation (Figure 2B): plants homozygous for QTL DM4.1.2 showed less sporulation than heterozygous plants, which in turn sporulated less than plants homozygous for absence of the QTL. Homozygous plants for QTL DM4.1.2 sporulated significantly more than NIL DM4.1, indicating that the DM4.1.2 introgression does not completely explain the loss-of-sporulation due to locus DM4.1. No significant differences were found in the population segregating for QTL DM4.1.2 regarding either chlorosis or necrosis.

In RHL DM4.1.3, significant differences (*p* < 0.05) were found regarding chlorosis (Figure 2C): plants homozygous for QTL DM4.1.3 showed significantly higher scores for chlorosis (indicating a smaller chlorotic leaf area with less intense chlorosis) than either heterozygous plants or plants homozygous for absence of DM4.1.3, whereas there were no significant differences in chlorosis between heterozygous plants and plants homozygous for absence of DM4.1.3, indicating a recessive effect of subQTL DM4.1.3 regarding chlorosis. Homozygous plants were not significantly more chlorotic compared to NIL DM4.1, indicating that subQTL DM4.1.3 fully explained the anti-chlorosis effect of QTL DM4.1. Plants homozygous for subQTL DM4.1.3 sporulated significantly less than plants homozygous for absence of DM4.1.3, indicating that subQTL DM4.1.3 also partially contributes to loss-of-sporulation, additional to its effect on chlorosis. Heterozygous individuals had spoulation scores in between those of plants homozygous for presence and plants homozygous for absence of QTL DM4.1.3, but were not significantly different to either of them.

Neither subQTL DM4.1.2 nor subQTL DM4.1.3 were found to have an effect on necrosis, as plants in both RHLs were not significantly different from susceptible control HS279, regardless of the presence of the subQTL (Figures 2B,C). An attempt was made to develop an RHL segregating for subQTL DM4.1.1, but no effect of presence of the introgression was found (Supplementary Figure 3), which might be explained by the fact that the segregating introgression in this family did not completely cover the previously detected DM4.1.1 interval (Supplementary Figure 3A).

### Transcriptomics Indicates That subQTL DM4.1.1 and/or DM4.1.2 Are Associated With Increased Differential Gene Expression Upon *P. cubensis* Inoculation, in Contrast to subQTL DM4.1.3

**FIGURE 3:**
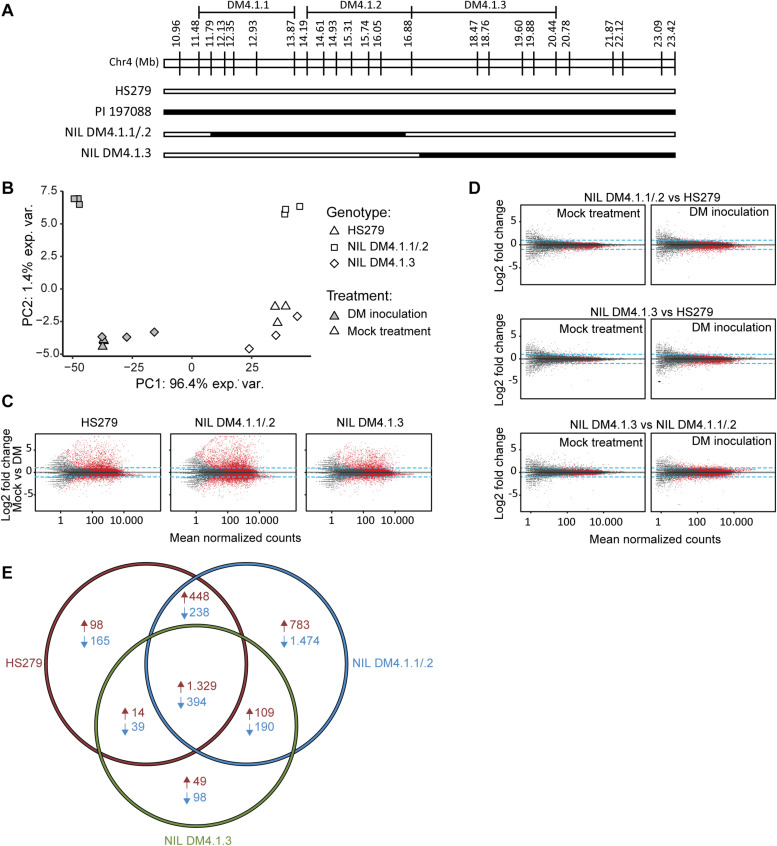
Transcriptome analysis of near-isogenic lines (NILs) with either subQTL DM4.1.2 or DM4.1.3. Analysis of transcriptome data from leaves of three cucumber genotypes (HS279, susceptible, and NILs DM4.1.2 and DM4.1.3, both partially resistant) 3 days post *P. cubensis* inoculation or mock control, with three independent samples per genotype × treatment combination. **(A)** Individuals homozygous for partial introgressions corresponding to subQTL DM4.1.1 and DM4.1.2 (NIL DM4.1.1/.2) or DM4.1.3 (NIL DM4.1.3) were selected to develop NILs. Bars represent the allele of genotypes at marker locations on the DM4.1 interval. Black bars indicate the PI 197088 allele and white bars indicate the HS279 allele. **(B)** Principal component analysis of transcriptome data. **(C)** MA plots for pairwise differential expression analysis contrasts between mock-treated and *P. cubensis* inoculated samples. Each point represents a detected gene. The *X*-axis represents the mean normalized counts per gene under both conditions, whereas the *Y*-axis represents the log_2_ fold change in *P. cubensis* inoculated samples compared to mock-treated samples. Differentially expressed genes (adjusted *p* < 0.05) are represented in red. Blue lines represent a twofold change threshold. **(D)** MA plots for pairwise differential expression analysis contrasts between genotypes under mock-treated (left column) or *P. cubensis* inoculated (right column) conditions. **(E)** Venn diagram representing differentially expressed upregulated (in red) and downregulated (in blue) genes in *P. cubensis* inoculated samples compared to mock-treated samples. Differentially expressed genes are here defined as statistically significant (adjusted *p* < 0.05) and >2-fold up- or downregulated.

Two homozygous individuals were selected for selfing to develop NILs, one (NIL DM4.1.1/.2) with an introgression corresponding to both subQTL DM4.1.1 and DM4.1.2, the other (NIL DM4.1.3) with an introgression corresponding to subQTL DM4.1.3 (Figure 3A). RNA was isolated from leaves of both NILs as well as from susceptible parent HS279, 3 days post inoculation with *P. cubensis* or a mock treatment, with three biological replicates. RNAseq yielded ca. 50M clean, trimmed 100 bp paired-end reads per sample, of which ca. 90% mapped to the cucumber reference genome (Chinese Long 9930 v2; Huang et al., 2009; Li et al., 2011). Raw sequencing data were deposited to NCBI SRA under accession number PRJNA544259.

Principal component analysis (PCA) of the RNAseq data revealed that the treatment (*P. cubensis* inoculation versus mock treatment) accounted for 96.4% of the observed variance in gene expression (Figure 3B). A genotype effect accounted for 1.4% of the observed variance, which separated NIL DM4.1.1/.2 from the other two genotypes. Biological replicates within each of the six genotype-treatment combinations clustered together in the PCA plot, although there was some variation between biological replicates of genotype NIL DM4.1.3 under both treatments (Figure 3B). Differential gene expression was determined based on an adjusted *p*-value < 0.05 and a fold-change > 2 (Supplementary Tables 2–4). Pairwise contrasts were established between treatments (Figure 3C), as well as between genotypes (Figure 3D). Consistent with greater PCA separation based on treatment, many more genes were differentially expressed in treatment comparisons (Figure 3C) than in genotype comparisons (Figure 3D). Additionally, more genes were differentially expressed between genotypes after *P. cubensis* inoculation (Figure 3D, right column) than after mock-treatments (Figure 3D, left column).

Cross-listing of differentially expressed genes due to the treatment effect in the three genotypes revealed that many more genes were uniquely up- and downregulated in NIL DM4.1.1/.2 compared to both NIL DM4.1.3 and the susceptible control HS279 (Figure 3E). In order to investigate whether specific biological processes were differentially influenced in NILs DM4.1.1/2 or DM4.1.3, a GO-term enrichment was performed on DEGs in both genotypes (Supplementary Table 5).

### Genes Upregulated by subQTL DM4.1.2 and/or DM4.1.1 Include Defense Pathway Genes

To test whether subQTL DM4.1.1 and DM4.1.2 on the one hand and DM4.1.3 on the other hand differentially influence defense pathways, expression patterns of cucumber homologs of known defense pathway genes were studied in more detail. Cucumber homologs of SA-inducible genes *PR1*, *PR2*, *EDS1*, *PAD4*, and *NPR1* as well as JA-inducible genes *PR3*, *PGIP2*, and *RST1* were identified by BLAST searches of the published *A. thaliana* protein sequences against the translated cucumber reference genome (Chinese Long 9930 v2).

**FIGURE 4:**
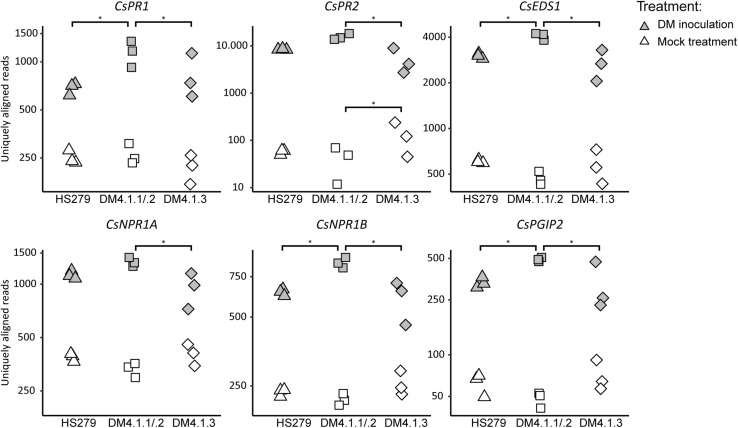
Expression analysis of defense pathway genes in the susceptible genotype HS279, a NIL with the sporulation reducing subQTL 4.1.2, and a NIL with the cholorosis reducing subQTL 4.1.3. Expression data of cucumber homologs of known defense pathway genes were extracted from the RNAseq dataset, and plotted per sample on a logarithmic scale. Asterisks represent statistically significant differences between genotypes (adjusted *p* < 0.05). For all genes, inoculation induced significant upregulation in all genotypes (*p* < 0.05). SubQTL 4.1.2 led to significantly higher expression of several defense pathway genes compared to subQTL 4.1.3, or the susceptible genotype HS279.

All examined defense pathway genes were significantly upregulated in *P. cubensis* inoculated plants compared to mock-treated plants for all three genotypes (with the exception of *CsPR3A*) (Figure 4 and Supplementary Figure 4). Generally, there were no significant differences in defense pathway gene expression between the genotypes in mock-treated plants (with the exception of *CsPR2*).

However, in *P. cubensis* inoculated plants, the mentioned defense pathway genes were generally higher expressed in NIL DM4.1.1/.2 compared to the other genotypes: Five out of the 13 studied defense genes were significantly higher expressed in NIL DM4.1.1/.2 compared to HS279, and eight out of the 13 genes were significantly higher expressed in NIL DM4.1.1/.2 compared to NIL DM4.1.3 (Figure 4 and Supplementary Figure 4), indicating that subQTL DM4.1.1 and/or DM4.1.2 are likely to harbor a causal gene triggering defense responses. In contrast, there were no significant differences in expression between NIL DM4.1.3 and HS279 for any of the 13 defense pathway genes, indicating that the causal gene underlying subQTL DM4.1.3 does not upregulate defense pathway genes.

### Fine-Mapping and Identification of Candidate Genes for subQTL DM4.1.2 for Reduced Sporulation

**FIGURE 5:**
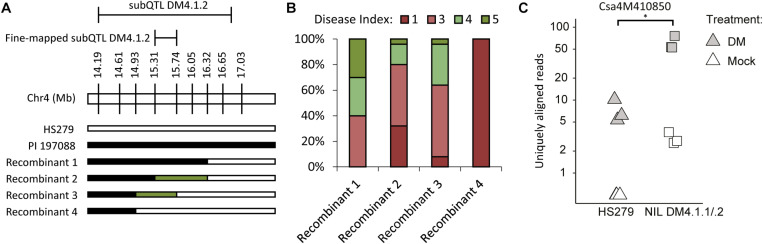
Fine-mapping and candidate gene expression subQTL DM4.1.2. **(A)** Screening of individuals derived from the mapping population allowed identification of additional informative recombinants within the DM4.1.2 interval. Bars represent genotypes at marker locations. Black bars indicate the PI 197088 allele, white bars indicate the HS279 allele, and green bars represent heterozygosity. **(B)** Populations were developed by self-fertilization of the recombinants described in A. 10–25 seedlings of each of the four populations was sown and used for a disease test. Sporulation was scored at 14 dpi on a scale from 1 to 9 as described before. Stacked bars represent the distribution of disease phenotypes in each of the four populations. **(C)** Expression data of gene Csa4M410850, encoding an *RLK* gene within the fine-mapped interval of DM4.1.2 interval, was extracted from the RNAseq dataset and plotted per sample on a logarithmic scale. No expression of this gene was detected in any of the mock-treated samples of the susceptible genotype HS279, but for visualization purposes, a 0.5 pseudocount was added. The asterisk denotes a significant difference between both genotypes in *P. cubensis* inoculated samples (adjusted *p* < 0.05). Differences between mock-treated and *P. cubensis* inoculated samples were also statistically significant (adjusted *p* < 0.05).

To narrow down the subQTL DM4.1.2 interval, additional plants were selected having recombinations within the interval, and lacking subQTL DM4.1.3 (Figure 5A). Four recombinants were selected, which together allowed fine-mapping of subQTL DM4.1.2 to the interval Chr4:15.309.857-15.738.683 (Figure 5A), containing 40 predicted genes in the cucumber reference genome (Chinese Long 9930 v2). These recombinants were selfed in order to create populations, which were tested for DM resistance (Figure 5B). One family derived from a recombinant homozygous for the PI197088 allele in this interval (recombinant 1) was sporulating relatively little, whereas a family derived from a recombinant homozygous for the HS279 allele in this interval (recombinant 4) was heavily sporulating. Two families derived from recombinants with heterozygous alleles at this interval (recombinants 2 and 3) were segregating for sporulation.

In order to identify the causal gene for subQTL DM4.1.2, we investigated the expression of these 40 genes in our RNAseq dataset (Supplementary Table 6). No genes were differentially expressed between the two genotypes in mock-treated samples, whereas only one gene (Csa4M410850) was differentially expressed between genotypes in *P. cubensis* inoculated samples. We found that Csa4M410850 was not detected at all in mock-treated HS279 plants, and at a very low level in mock-treated NIL DM4.1.1/.2 plants. However, the gene was upregulated in both genotypes after *P. cubensis* inoculation, although to a ca. tenfold higher level in NIL DM4.1.1/.2 compared to HS279 (Figure 5C). As Csa4M410850 has been annotated as an *RLK* gene, which are frequently involved in defense signaling, we selected it as an interesting candidate gene for subQTL DM4.1.2.

Additionally, we identified four non-synonymous polymorphisms (SNPs) in our RNAseq dataset between HS279 and NIL DM4.1.1/.2 in coding sequences of three predicted genes within the fine-mapped interval Chr4:15.309.857-15.738.683 (Supplementary Table 7). One of these genes was Csa4M410830, which was also annotated as an *RLK* gene, forming a cluster of *RLK* genes together with Csa4M410850. Based on the annotation of Csa4M416480 and Csa4M416990, we do not consider these two genes as candidates.

### Genomic Analysis of the *RLK* Locus in QTL DM4.1.2 Indicates a Structural Variation

As we selected two *RLK* genes (Csa4M410830 and Csa4M410850) as the most likely candidates for subQTL DM4.1.2, we decided to study the genomic context of these genes. To this end, we visually inspected the alignment of sequencing reads in the *RLK* locus (Ch4:15.413.000-15.435.000).

**FIGURE 6:**
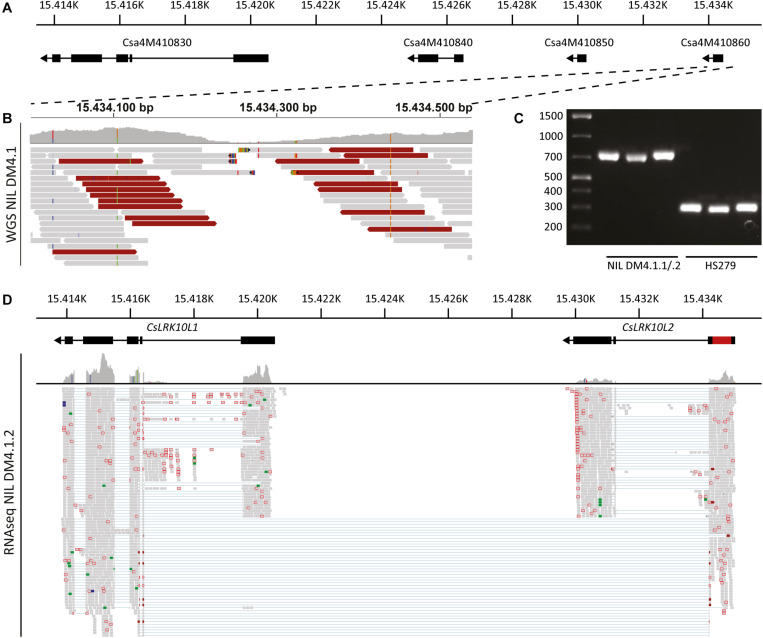
Genomic analysis of the *RLK* cluster DM4.1.2, indicating structural variation. **(A)** Predicted gene models of genes in the *RLK* cluster within the interval of subQTL DM4.1.2. Black boxes indicate predicted exons and lines represent predicted introns. Arrowheads indicate the orientation of the reading frame. Physical locations on chromosome 4 of the cucumber reference genome (Chinese Long 9930 v2) are indicated. **(B)** Whole genome sequencing reads of NIL DM4.1 aligning to predicted gene Csa4M410860 are visualized using the Integrative Genomics Viewer (IGV). A coverage graph is given above the aligned reads. Reads pairs with larger than expected or smaller than expected insert sizes are indicated in dark red and dark blue, respectively. **(C)** PCR was performed using primers designed to amplify the predicted gene *Csa4M410860* on DNA samples isolated from the partially resistant NIL DM4.1.1/.2 and the susceptible recurrent parent HS279. **(D)** RNAseq reads from *P. cubensis* inoculated NIL DM4.1.1/.2 were re-aligned to the *RLK* cluster including the 551 bp insertion found in the predicted gene *Csa4M410860*. Gene models of *CsLRK10L1* (Csa4M410830) and *CsLRK10L2* (novel gene consisting of both Csa4M410850 and Csa4M410860) are indicated above, including the 551 bp insertion in red. Numbers indicate physical location on chromosome 4 of the cucumber reference genome (Chinese Long 9930 v2).

The cluster of predicted *RLK* genes in the DM4.1.2 interval consists of three genes: Csa4M410830, Csa4M410840, and Csa4M410850 (Figure 6A). Visual inspection of aligned RNAseq reads revealed that reads aligning to the Csa4M410850 locus actually form a longer transcript than predicted, consisting of three exons, one of which was predicted to be a separate gene (Csa4M410860) without any annotation (Supplementary Figure 5A). The first *RLK* gene in the cluster (Csa4M410830) was abundantly transcribed, whereas there were no indications of transcription of Csa4M410840 in our RNAseq data (Supplementary Figure 5A).

To clarify the *RLK* locus structure, we performed whole genome sequencing (WGS) of NIL DM4.1. Sequencing reads were aligned to the cucumber reference genome (Chinese Long 9930 v2). Visual inspection of WGS reads aligning to the *RLK* locus indicated a structural variation in Csa4M410860, characterized by a local, drastic decrease in coverage at the interval Chr4:15.434.200-15.434.350 and deviating insert sizes of mate pairs flanking this interval (Figure 6B and Supplementary Figure 5B).

To validate the suspected structural variation in Csa4M410860, primers were developed flanking the locus. PCR on DNA isolated from susceptible recurrent parent HS279 amplified the ca. 250 bp product identical to the reference genome, whereas the PCR product from NIL DM4.1.1/.2 was ca. 700 bp longer (Figure 6C). Sanger sequencing of the amplicon revealed the presence of a 551 bp insertion in NIL DM4.1.1/.2 compared to HS279 and the reference genome. Realignment of NIL DM4.1.1/.2 RNAseq reads to the *RLK* cluster including the 551 bp insertion demonstrated the presence of an apparently intact 1.905 bp long gene, comprising Csa4M410850 and Csa4M410860 including the 551 bp indel (Figure 6D). The sequence of this annotated gene was deposited to NCBI GenBank [MK936607].

### The Presumably Causal *RLK* Gene Is Present in One Quarter of Resequenced Accessions

In order to see how the 551 bp indel which we presume to be causal for subQTL DM4.1.2 is distributed in the cucumber germplasm, we performed an *in silico* search for either the 551 bp deletion allele or the 551 bp insertion allele in genome resequencing data of a collection of 115 divergent cucumber accessions (Qi et al., 2013) (Supplementary Table 8). We found that we could identify the allele containing the 551 bp insertion (and therefore presumably the functional *RLK* gene) in 32 out of the 115 accessions. Interestingly, the majority (22 out of 30) of the “Indian” cucumber accessions, as defined by Qi et al. (2013), do contain the 551 bp insertion, whereas a minority (10/29) of the “Eurasian” cucumber accessions share this allele. It was not detected in any of the 37 “East Asian” or 19 “Xishuangbanna” accessions.

### Annotation of *RLK* Genes in the DM4.1.2 Locus Based on Sequence Analysis Reveals That Both Genes Belong to *Leaf Rust Kinase 10-Like* Families 1 and 2

**FIGURE 7:**
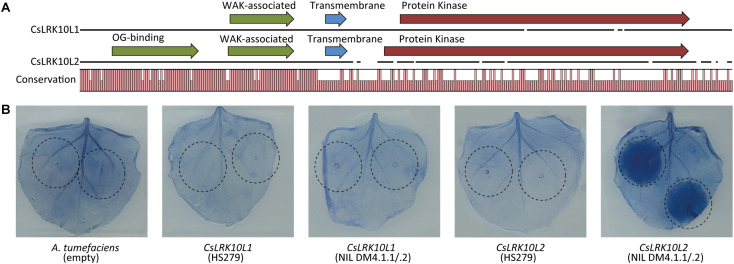
Functional characterization of *CsLRK10L* genes. **(A)** Multiple protein sequence alignment of predicted proteins encoded by *CsLRK10L1* and *CsLRK10L2*. A graph indicates conservation per amino acid. Pfam domains as identified by InterProScan v5.27 are indicated by arrows. **(B)** Alleles of *CsLRK10L1* and *CsLRK10L2* cloned from cucumber genotypes HS279 and NIL DM4.1.1/.2 were transiently expressed in *Nicotiana benthamiana* leaves by agro-infiltration (infiltrated leaf areas are indicated). Empty *A. tumefaciens* cultures were used as a negative control. *CsLRK10L2* cloned from NIL DM4.1.1/.2 consistently triggered a necrotic reaction in >20 individual plants. In order to visualize necrotic tissue, leaves were stained using trypan blue.

We found that the N-terminal parts of the predicted proteins of both *RLK* genes in the DM4.1.2 locus were conserved (>90% identical), whereas the C-terminal parts were less conserved (<30% identical) (Figure 7A). InterPro domain annotation of the predicted protein sequences indicated that the conserved, N-terminal parts of both proteins contain predicted WAK-associated domains (IPR032872), characteristic for *Wall-Associated RLK* (*WAKL*) genes (Figure 7A). The novel *RLK* gene also contained a predicted oligogalacturonan-binding domain (IPR025287), which is also commonly found in *WAKL* genes. The non-conserved C-terminal part of the proteins both contained predicted transmembrane helices and protein kinase domains (IPR000719), but whereas the predicted active site of the protein encoded by *RLK* gene Csa4M410830 contained the conserved arginine-aspartate (RD)-motif, this motif was lost in the protein encoded by the novel *RLK* gene.

To find more evidence for the function of the identified *RLK* gene, we compared the gene family in cucumber with the better functionally annotated *RLK* gene family in *A. thaliana*. We performed BLASTp queries against both translated genomes of cucumber (Chinese Long 9930 v2) and *Arabidopsis* (TAIR 10), using the oligogalacturonan-binding, WAK-associated, and Protein Kinase domains of both genes. As was expected, BLAST results using the conserved extracellular domains of both proteins identified the same set of homologs. There was, however, no overlap between the BLAST output regarding the protein kinase domains of both *RLKs* (Supplementary Table 9). Kinase domain homologs of the protein encoded by *RLK* Csa4M410830 belong to *RLK* subfamily *LRK10-like 1*, according to the nomenclature proposed by Shiu and Bleecker (2003), whereas many of the kinase domain homologs of the protein encoded by the novel *RLK* gene belong to subfamily *LRK10-like 2*. The majority of the homologs of both genes regarding the extracellular domains also belonged to the *LRK10-like 1* and *2* subfamilies. Therefore, we will further refer to these genes as *CsLRK10L1* (Csa4M410830) and *CsLRK10L2* (the newly identified *RLK* gene corresponding to Csa4M410850 and Csa4M410860).

In order to verify whether identified homologs of *CsLRK10L1* and *CsLRK10L2* in cucumber and *Arabidopsis* had predicted oligogalacturonan-binding and/or WAK-associated domains, we scanned the complete predicted proteomes of cucumber (Chinese Long 9930 v2) and *Arabidopsis* (TAIR 10) using InterProScan v5.27 for presence of oligogalacturonan-binding (IPR025287) and/or WAK-associated (IPR032872) domains. We identified 35 cucumber and 42 Arabidopsis proteins with such domains (Supplementary Table 10). Phylogenetic [maximum likelihood (ML)] trees were constructed based on the kinase domains of BLAST-identified homologs of both *CsLRK10L1* (Supplementary Figure 6A) and *CsLRK10L2* (Supplementary Figure 6B), as well as on the extracellular domains of all identified proteins with OG-binding and/or WAK-associated domains (Supplementary Figure 6C).

As the gene identifiers of predicted WAK*-*domain genes indicated that many of them were closely together on the cucumber and *Arabidopsis* genomes (i.e., consecutive gene IDs), we mapped the location of each of the WAK-domain genes on their respective genomes (Supplementary Figure 7) and found that indeed the majority of genes with predicted WAK domains were part of clusters in the genome.

### Characterizing the *RLK* Genes in the DM4.1.2 Locus Reveals That *CsLRK10L2* Can Trigger Necrosis

We cloned and sequenced both *CsLRK10L1* and *CsLRK10L2* from cDNA of genotypes HS279 and NIL DM4.1.1/.2 in order to verify the expression and predicted coding sequences of both genes. Sanger sequencing of both alleles of both *RLK* genes confirmed the expected sequences based on the assembled genes.

In order to functionally characterize the *RLK* genes, we transiently overexpressed both alleles of both genes in leaves of *N. benthamiana*. Three days post infiltration, a necrotic reaction was observed in the leaf area infiltrated with the NIL DM4.1.1/.2 allele of *CsLRK10L2*, but not in the parts of the leaves infiltrated with the HS279 allele of *CsLRK10L2*, nor with either allele of *CsLRK10L1*. Leaves were stained with trypan blue to visualize the necrotic tissue (Figure 7B).

## Discussion

### QTL DM4.1 Consists of Multiple subQTL Contributing to DM Resistance

Our results confirm the presence of QTL DM4.1 from resistant donor PI 197088, conferring a partial level of DM resistance. Whereas introgression of this QTL in a susceptible background genotype on its own only confers partial DM resistance, it could well be combined with other QTL in order to develop DM resistant cucumber cultivars. Furthermore, our results also show that there are at least two (and probably three or more) subQTL underlying QTL DM4.1, each with different quantitative effects. We identified recombinants between subQTL DM4.1.2, conferring anti-sporulation, and DM4.1.3, conferring anti-chlorosis, showing that both traits of QTL DM4.1 are controlled by separate genes. Furthermore, we postulate the existence of a third subQTL, DM4.1.1, conferring anti-necrosis. However, we failed to identify recombinants between DM4.1.1 and DM4.1.2 which unambiguously prove the existence of this anti-necrosis subQTL, and therefore used a NIL comprising both postulated subQTL (NIL DM4.1.1/2) for our subsequent work. Various factors might influence our inability to unequivocally identify a subQTL for anti-necrosis, even though plants with a full DM4.1 introgression do show an anti-necrosis effect (Figures 2B,C), such as environmental effects on necrosis which cause inter-experimental variation; or an epistatic interaction between subQTL.

Previously, several groups have mapped DM resistance inherited from accession PI 197088. Whereas the previous publications describing a single QTL on chromosome 4 all mapped QTL for DM resistance in populations also segregating for other loci in the genome, we developed populations segregating for loci in the DM4.1 interval only, in a homozygous, susceptible, background, aiming at greater resolution. Furthermore, we distinguished different symptoms separately (i.e., chlorosis, sporulation, and necrosis) whereas other groups mapping QTL for DM resistance in PI197088 used “overall” DM disease indices. The combination of these two factors enabled us to discover multiple separate subQTL in the DM4.1 locus.

Recently another group also scored their segregating populations using multiple disease phenotype criteria, i.e., yellowing (chlorosis), collapsing (necrosis), and “general impression” (Wang et al., 2018). These populations were derived from other sources of resistance, i.e., cucumber genotypes Gy14 and WI 2757. They found that whereas a delay in chlorosis was highly correlated with the score based on general impression, there was no strong correlation between scores for yellowing and collapsing, indicating that anti-chlorosis and anti-necrosis in these populations had a different genetic basis. Both this study and our results demonstrate that it can be wise to score multiple aspects of disease progression separately, as this might give a more complete picture of disease resistance, potentially enabling the identification of QTL which might be overlooked by a simpler scoring.

### SubQTL DM4.1.1 and/or DM4.1.2 Upregulate Defense Pathway Genes Upon Inoculation, in Contrast to subQTL DM4.1.3

Recently, Burkhardt and Day (2016) investigated transcriptomic trends of resistant cucumber accession PI 197088 and a susceptible control (cv. Vlaspik) in a time course after inoculation with *P. cubensis*. They found that thousands of genes were differentially expressed between mock-treated and *P. cubensis* inoculated plants, and that this response was stronger and faster in PI 197088 compared to the susceptible control. As resistance in PI 197088 is highly polygenic (Yoshioka et al., 2014; Wang et al., 2017; Li et al., 2018), we were interested to find out which transcriptomic changes could be attributed to the QTL we are studying.

Our RNAseq results on NILs with either subQTL DM4.1.1 and DM4.1.2 or with DM4.1.3, and the susceptible parent HS279, indicated that in agreement with the previous findings (Burkhardt and Day, 2016), *P. cubensis* inoculation drastically alters gene expression (Figures 3B,C). In contrast, gene expression differences between the three studied genotypes was rather subtle (Figures 3B,D), as was to be expected based on the similar genetic background of these genotypes. However, by comparing the differentially expressed genes due to *P. cubensis* inoculation in the three genotypes, we found that 56.7% of all downregulated genes and 27.7% of all upregulated genes were uniquely up/downregulated in NIL DM4.1.1/.2, whereas only low amounts of genes were uniquely up- or downregulated in HS279 and NIL DM4.1.3 (Figure 3E). This indicated that the partial resistance conferred by subQTL DM4.1.1 and/or DM4.1.2 is associated with rather large differences in gene expression, in contrast to partial resistance conferred by subQTL DM4.1.3. More detailed analysis of cucumber homologs of known SA- as well as JA-induced defense pathway genes indicated that several of these were stronger upregulated in NIL DM4.1.1/.2 compared to the other two genotypes after *P. cubensis* inoculation (Figure 4), implying that DM4.1.1/.2-associated resistance is linked to known defense pathways. It is at present not possible to ascertain whether the observed upregulation of defense pathway genes is caused by either subQTL DM4.1.1, DM4.1.2, or both, as this would require transcriptomic studies on NILs with either subQTL in absence of the other.

The finding that defense pathway genes are upregulated in NIL DM4.1.1/.2 is in line with our identification of an *RLK* gene as a potentially causal gene for subQTL DM4.1.2, as RLK proteins often trigger defense responses, and additionally overexpression of this particular gene triggered a necrotic defense response in *N. benthamiana*. It is well possible that a causal gene underlying QTL DM4.1.1 also contributes to the increase of defense gene expression observed in NIL DM4.1.1/.2, and it might be assumed that such a gene would then have a function similar to that of the *RLK* gene we identified as candidate for subQTL DM4.1.2, although more fine-mapping studies are required to identify this gene, as well as transcriptomic studies of a NIL with subQTL DM4.1.1 in absence of DM4.1.2. In contrast, DM4.1.3-associated resistance apparently depends on other mechanisms, as no significant differences in defense gene expression were found between NIL DM4.1.3 and susceptible parent HS279. As the anti-chlorosis effect of subQTL DM4.1.3 inherits as a recessive gene, we anticipate that the causal gene underlying this subQTL might be a loss-of-function allele of a susceptibility (S) gene, rather than an active component of plant defense such as an *RLK* gene. S-genes are known to have diverse functions, ranging from negative regulation of defense responses to metabolism and transport of nutrients required by pathogens (van Schie and Takken, 2014). More fine-mapping studies are underway to identify the causal gene underlying subQTL DM4.1.3.

### The *RLK*-Gene CsLRK10L2 Is Likely the Causal Gene for subQTL DM4.1.2

By combining fine-mapping (Figures 5A,B) with differential gene expression analysis (Supplementary Table 6 and Figure 5C), we selected the *CsLRK10L2* gene (Csa4M410850) as the most likely candidate gene for subQTL DM4.1.2. This fitted our previous observation that resistance conferred by this subQTL is associated with increased expression of defense pathway genes (Figure 4), as *RLK* genes are commonly known to be frequently involved in pathogen signaling (Tang et al., 2017).

Interestingly, the 551 bp insertion that was found in the DM4.1.2 allele of *CsLRK10L2* led to a predicted gene of normal size. It is likely that the DM4.1.2 allele represents the functional allele of the *RLK* gene, whereas the deletion-allele of HS279, which is also present in the cucumber reference genome Chinese Long 9930, represents a loss-of-function mutation. The question is then why domesticated cucumber genotypes such as HS279 and Chinese Long 9930 have lost this gene, as this would decrease their resistance to *P. cubensis*. Our *in silico* identification of both alleles of *CsLRK10L2* in a collection of 115 resequenced cucumber accessions (Supplementary Table 8) showed that both alleles are present in “Indian” type cucumber, including roughly half (seven out of 13) of the wild *C. sativus* var. *hardwickii* accessions. This does support the notion that the 551 bp insertion allele could represent the ancestral state of the gene, whereas the 551 bp deletion allele represents a loss of function allele, which apparently became fixed in East Asian germplasm and became the major allele in Eurasian germplasm.

Based on the presence of WAK-like and oligogalacturonan-binding domains in the extracellular domains of *CsLRK10L2* (Figure 7A), we speculate that this gene might be involved in signaling of loss of cell wall integrity, similar to *WAKL* genes in which such domains are usually found (Kohorn, 2001, 2016; Verica and He, 2002). Many *WAKL* genes have been found to contribute to quantitative resistance against various fungal and bacterial pathogens in multiple plant species (Diener and Ausubel, 2005; Johansson et al., 2006; Li et al., 2009b; Rosli et al., 2013; Hurni et al., 2015; Zuo et al., 2015; Delteil et al., 2016). Our working hypothesis is that *CsLRK10L2* contributes to quantitative disease resistance against *P. cubensis* through oligogalacturonan perception. This fits with the observation that the gene is able to trigger necrosis in *N. benthamiana* leaves without supplying an external ligand, as oligogalacturonan is normally present in small concentrations due to plant-encoded polygalacturonases.

### Other Putative Candidate Genes for subQTL DM4.1.2

We selected *CsLRK10L2* as the most likely candidate gene for subQTL DM4.1.2. However, there were other potential candidate genes as well. Our results showed that there were non-synonymous SNPs in three genes within the DM4.1.2 interval (Supplementary Table 7).

One of those genes was *CsLRK10L1* (Csa4M410830), located in the same cluster as *CsLRK10L2*. Contrary to our findings regarding *CsLRK10L2*, overexpression of *CsLRK10L1* had no effect in *N. benthamiana*. However, we cannot rule out the possibility that this gene could have an effect on DM resistance in cucumber.

The second gene in the interval with a non-synonymous SNP was Csa4M416480. This gene has high homology (82% identical amino acid sequences) to the *Arabidopsis CBR1* gene, which is involved in fatty acid desaturation in developing seeds and male gametophytes. *cbr1* loss of function mutants were found to have defects in male fertility, seed setting, and seed germination whereas vegetative growth was unaffected (Wayne et al., 2013), but to our knowledge, no effects of *CBR1* or related genes on pathogen resistance are known. Additionally, the substitution in the encoded protein in the NIL DM4.1.1/.2 allele of this gene was conservative, as valine and isoleucine have rather similar physiochemical properties.

The third gene in the interval with (two) non-synonymous mutations is Csa4M416990, encoding a cucumber homolog (53% identical amino acid sequences) of *Arabidopsis* F-box gene *SKIP24*. F-box proteins are part of the SCF complex, which is involved in protein ubiquitination leading to subsequent proteolysis. The F-box protein subunit of this complex is thought to grant substrate specificity to the complex and as such F-box genes form a very diverse and abundant gene family (Kipreos and Pagano, 2000; Risseeuw et al., 2003). The specificity of *SKIP24* and closely related F-box proteins is to our knowledge unknown, and no mutant phenotypes are available, leading us to focus on the *CsLRK10L* genes in this publication instead. However, Wang (2017) reported fine-mapping QTL DM4.1 from cucumber accession WI 7120 (PI 330628) to an 82 kb interval, containing 13 predicted genes, including *CsSKIP24* (Csa4M416990) as the most likely candidate gene. Whereas the causal genes of QTL DM4.1 from PI 330628 and DM4.1.2 from PI 197088 are not by definition allelic, we found mutations in the same gene in our genotype. It is in principle possible that subQTL DM4.1.2 in PI 197088 has two causal genes, one of which is shared with PI 330628. Functional studies regarding candidate genes *CsLRK10L2* and *CsSKIP24*, e.g., by complementation in DM susceptible cucumber, are needed in order to verify whether either or both genes are involved in DM resistance. Furthermore, additional fine-mapping experiments in our PI 197088 derived NILs might enable us to verify whether this subQTL can either be further divided in two subQTL, or delimited to a region excluding one or both of the candidate genes. However, such experiments were outside the scope of the current publication.

### Phylogenetic Analysis of *CsLRK10L* Genes Reveals Patterns of Domain Reshuffling

In a phylogenetic analysis of the predicted extracellular domains of RLK proteins of cucumber and *A. thaliana* with OG-binding and/or WAK-associated domains, we found that the extracellular domains of *CsLRK10L1* and *CsLRK10L2* were close homologs of one another, being more closely related to one another than to any other gene (Supplementary Figure 6C). This indicates that the two genes likely arose due to tandem duplication, as is often the case for *RLK* genes. However, our results showed that regarding the intracellular kinase domains, each of the two genes has a distinct set of homologs (Supplementary Figures 6A,B), indicating a unique evolutionary history for both of the two genes. Apparently, the extracellular domain of one ancestral gene, encoded by the first exon, was duplicated, but was subsequently fused to an alternative intracellular kinase domain. Previously, it was reported that in general *RLKs* with similar extracellular domains also have similar kinase domains, with the exception of *LRK10L*, *CrRLK1-like*, and S-domain *RLKs*, which can have several different kinase domains (Shiu and Bleecker, 2003).

### Several Kinase Domain Homologs of CsLRK10L2 Are Involved in Disease Resistance

*Arabidopsis* homologs of the *CsLRK10L2* kinase domain included eight genes not previously described in literature, as well as five previously described genes, some of which were found to be involved in plant–pathogen interactions. One *CsLRK10L2* kinase domain homolog was the *Arabidopsis LRK10L2* gene, which gives its name to the *LRK10L2* subfamily of *RLK* genes. This gene was named after the wheat *LRK10* gene due to sequence homology of the extracellular domain (Shiu and Bleecker, 2003). The wheat *LRK10* gene was found to be a candidate gene for the *lr10* locus, contributing to resistance to leaf rust caused by *Puccinia recondita* (Feuillet et al., 1997). Another *CsLRK10L2* homolog is *Arabidopsis PR5K*, which has an extracellular domain homologous to pathogenesis related PR5 proteins (Wang et al., 1996), and overexpression of which in creeping bentgrass (*Agrostis palustris*) led to increased resistance to *Sclerotinia homoeocarpa*. Yet another homolog in the *CsLRK10L2* clade is *SNC4*, an *RLK* with two predicted extracellular glycerophosphoryl diester phosphodiesterase domains. An auto-active mutant allele of *SNC4* obtained in an EMS screen had increased resistance against DM caused by *Hyaloperonospora arabidopsidis*, as well as elevated expression of SA-marker genes *PR1* and *PR2* and JA-marker gene *PDF1.2* (Bi et al., 2010). Finally, two other *CsLRK10L2* kinase domain homologs were *MDS3* and *MDS4*, which have malectin-like extracellular domains classifying them as *CrRLK1L* family members. CrRLK1L malectin-like domains are thought to bind to pectin in the cell wall, similar to WAK proteins, even though the sequences of CrRLK1L and WAK proteins are not very homologous (Feng et al., 2018). *MDS3* and *MDS4* were found to be involved in growth regulation under heavy metal ion stress (Richter et al., 2018).

### Conclusion and Future Perspectives

We have shown that QTL DM4.1 from PI 197088 consists of three subQTL, each with different effects on disease phenotype. One subQTL, DM4.1.2, caused a decrease in sporulation and was associated with increased expression of defense pathway genes. We further focused on this subQTL, and identified a candidate gene, *CsLRK10L2*. A 551 bp deletion, leading to a loss-of-function allele, was found in susceptible genotype HS279, similar to the reference genome Chinese Long 9930 v2. It was found that the intracellular kinase domain of the encoded protein was homologous to several *Arabidopsis* RLKs with known functions in plant defense against several unrelated pathogen. Furthermore, we found that heterologous overexpression of the gene in *N. benthamiana* triggered a necrotic response, in contrast to the susceptible allele. We consider the possibility that *CsLRK10L2* plays a role as a receptor of the DAMP oligogalacturonan, the breakdown product of pectin in the plant cell wall. More experimental work is needed to confirm whether CsLRK10L2 is indeed the causal gene for subQTL DM4.1.2, and if so, to identify the mechanisms by which this protein leads to increased DM resistance. Furthermore, three additional candidate genes were identified based on non-homologous polymorphisms between genotypes with and without the genotypes, of which *CsSKIP24* (Csa4M416990) stood out based on the finding that mutations in this gene also occurred in another DM resistant genotype (Wang, 2017).

Future work will hopefully lead to fine-mapping and identification of candidate genes for the other subQTL as well, especially regarding DM4.1.3 which had a recessive effect on both chlorosis as well as sporulation.

## Data Availability Statement

The datasets presented in this study can be found in online repositories. The names of the repository/repositories and accession number(s) can be found in the article/Supplementary Material.

## Author Contributions

FH, FB, and WV developed plant materials and performed QTL mapping and (fine)mapping. JB designed, performed, and analyzed the rest of the experiments, with valuable suggestions by HS and YB as well as assistance by FB and LL. JB drafted the manuscript. WV, RV, YB, and HS gave critical feedback on the draft manuscript. All authors read and approved the manuscript.

## Conflict of Interest

The authors declare that the research was conducted in the absence of any commercial or financial relationships that could be construed as a potential conflict of interest.

## Supplementary Material

Supplementary Figure 1 Representative pictures for DI Chlorosis, Sporulation and Necrosis. For each of the three scored traits, representative pictures are given of the spectrum of the DI classes from 1 (susceptible) to 9 (resistant).

Supplementary Figure 2 Genotypic and phenotypic data of 19 recombinant families derived from a QTL isogenic introgression line. 19 F3BC3S3 individuals with recombination events within the DM4.1 locus were selected and genotyped, and progeny of these 19 plants were evaluated in a disease assay. **(A)** Bars represent the allele of genotypes at marker locations on the DM4.1 interval. Black bars indicate the PI 197088 allele, white bars indicate the HS279 allele, green bar represents heterozygosity. Populations were developed by self-fertilization of the 19 recombinants described in **(A)**. 20 to 91 seedlings of each of the 19 populations were shown and used for a disease assay. Sporulation **(B)**, chlorosis **(C)** and necrosis **(D)** were scored at 14 dpi on a scale from 1-9 as described before. Stacked bars represent the distribution of disease phenotypes in each of the 19 populations.

Supplementary Figure 3 *P. cubensis* disease test on RHL DM4.1.1. Similarly as described for Figure 3, a family was developed segregating for a partial introgression corresponding to part of subQTL DM4.1.1. **(A)** Bars represent the allele of genotypes at marker locations on the DM4.1 interval. Black bars indicate the PI 197088 allele, white bars indicate the HS279 allele, green bar represents heterozygosity. **(B)** The RHL was inoculated with *P. cubensis*, chlorosis was scored at 7 dpi whereas sporulation and necrosis were scored at 12 dpi. Bars represent average phenotype scores on a 1-9 scale ranging from susceptible to resistant. Error bars indicate standard deviations. Bars with different letters indicate statistically significant differences (Kruskal-Wallis test, *p* < 0.05).

Supplementary Figure 4 Expression analysis of defense pathway genes. Expression data of cucumber homologs of known defense pathway genes were extracted from the RNAseq dataset, and plotted per sample on a logarithmic scale. Asterisks represent statistically significant differences between genotypes (adjusted *p* < 0.05). For all genes except *CsPR3A*, differences between treatments within genotypes were statistically significant (*p* < 0.05).

Supplementary Figure 5 RNAseq and WGS alignment *RLK* cluster. **(A)** RNAseq reads from *P. cubensis* inoculated NIL DM4.1.1/.2 aligning to the RLK cluster are visualized using the Integrative Genomics Viewer (IGV). Split reads are indicated with blue lines. A coverage graph is given above the aligned reads. **(B)** Whole genome sequencing reads of NIL DM4.1 aligning to the RLK cluster are visualized using the Integrative Genomics Viewer (IGV). A coverage graph is given above the aligned reads. Reads pairs with larger than expected or smaller than expected insert sizes are indicated in dark red and dark blue, respectively. Dotted lines denote the interval shown at greater resolution in Figure 6B, corresponding to predicted gene Csa4M410860.

Supplementary Figure 6 Phylogenetic analysis *CsLRK10L* genes. Homologs of the kinase domains of CsLRK10L1 **(A)** and CsLRK10L2 **(B)** were identified using BLASTp against cucumber and *Arabidopsis* translated reference genomes, and used to construct phylogenetic (maximum likelihood) trees. Cucumber and *Arabidopsis* homologs are indicated with green and red circles, respectively. Branches leading to homologs with predicted oligogalacturonan (OG) binding and/or WAK-associated domains are colored blue. **(C)** A phylogenetic (maximum likelihood) tree was constructed based on predicted OG-binding domains of *Arabidopsis* and cucumber proteins. Clades containing the majority of previously annotated WAK, WAKL and LRK10L proteins are indicated.

Supplementary Figure 7 Physical locations *WAKL/LRK10L* genes. Genomic positions of genes encoding proteins with predicted galacturonan-binding and/or WAK-associated domains were retrieved and visualized.

Supplementary Table 1 SNP markers used and QTL detected for the three traits Chlorosis, Sporulation and Necrosis in the DM4.1 mapping population. Physical locations of nine SNP markers used in QTL analysis and subsequent fine-mapping are given based on the cucumber reference genome (Chinese Long 9930 v2). Furthermore, flanking and peak markers for each of the QTL detected in Figure 1 are given.

Supplementary Table 2 Differentially expressed genes between genotypes, inoculated. Pairwise contrasts of differentially expressed genes (DEGs) between the three genotypes (NIL DM4.1.1/.2, NIL DM4.1.3 and HS279), comparing inoculated samples. Complete DEseq2 (v3.8) output is given.

Supplementary Table 3 Differentially expressed genes between genotypes, mock-treated. Pairwise contrasts of differentially expressed genes (DEGs) between the three genotypes (NIL DM4.1.1/.2, NIL DM4.1.3 and HS279), comparing mock-treated samples. Complete DEseq2 (v3.8) output is given.

Supplementary Table 4 Differentially expressed genes within genotypes. Pairwise contrasts of differentially expressed genes (DEGs) between mock treated and inoculated samples within each of the three genotypes (NIL DM4.1.1/.2, NIL DM4.1.3 and HS279. Complete DEseq2 (v3.8) output is given.

Supplementary Table 5 GO term enrichment. GO-term enrichment was performed on DEGs in genotypes NIL DM4.1.1/.2 and NIL DM4.1.3 (both up- and downregulated genes). Lists are given for GO-terms significantly enriched in either of the four DEG lists (up- and downregulated upon DM inoculation, in both NILs).

Supplementary Table 6 Differential gene expression analysis in fine-mapped DM4.1.2 interval. Expression data of genes within the physical interval of the fine-mapped subQTL DM4.1.2 were analyzed. Pairwise contrasts between genotypes NIL DM4.1.1/.2 and HS279 under both conditions as well as between both conditions for both genotypes are given. Both the Log_2_ of the fold change and the adjusted *P* value are given. Additionally, physical locations and annotations per gene are indicated.

Supplementary Table 7 Polymorphisms in fine-mapped DM4.1.2 interval. SNPs and indels in the fine-mapped DM4.1.2 interval were determined using the SAMtools mpileup command, and annotated using SnpEff. Non-synonymous SNPs are given.

Supplementary Table 8 *In silico* detection 551 bp insertion allele *CsLRK10L2* in a collection of 115 resequenced cucumber accessions. The collection of 115 resequenced cucumber accessions (Qi et al., 2013) was probed *in silico* for reads containing evidence for presence or absence of the 551 bp insertion allele identified in NIL DM4.1. For each individual in which the 115 bp insertion allele could be identified, SRR number of the resequencing data, PI/CGN number of the accession, accession names, country of origin and “type” (Eurasia, East Asia, India or Xishuangbanna) is defined by Qi et al. (2013) is given.

Supplementary Table 9 BLAST output CsLRK10L protein domains. Galacturonan binding (GUB), WAK-associated (WAK) and protein kinase domains of CsLRK10L1 and CsLRK10L2 were used as BLASTp queries against translated *Arabidopsis* and cucumber genomes. Tabular BLAST output is given.

Supplementary Table 10 InterProScan analysis WAK domains. Predicted proteins in the translated cucumber and *Arabidopsis* genomes were scanned using InterProScan v 5.27 for presence of galacturonan binding and WAK-associated domains. Start and end locations of predicted domains are indicated.

Supplementary Table 11 Predicted cucumber defense pathway genes. Cucumber orthologues of known *Arabidopsis* defense pathway genes were identified by BLASTp search followed by phylogenetic analysis. When multiple orthologues were found, capital letters were added as a suffix.
