# Supplementary figures and images for: Analysis of QTL DM4.1 for Downy Mildew Resistance in Cucumber Reveals Multiple subQTL: A Novel RLK as Candidate Gene for the Most Important subQTL
Source: Front Plant Sci. 2020 Oct 22;11:569876. doi: 10.3389/fpls.2020.569876 (PMC7649820; doi:10.3389/fpls.2020.569876)

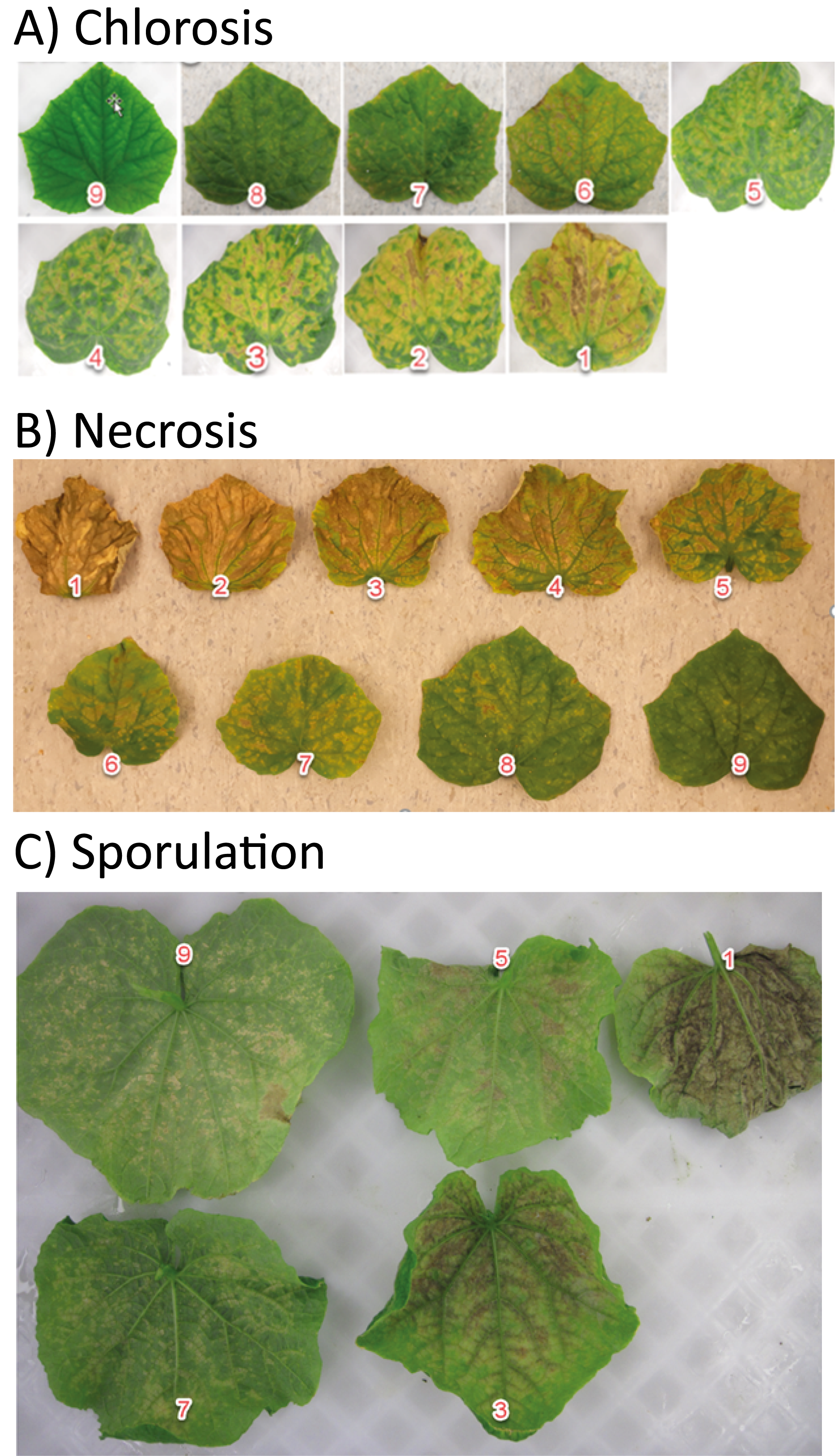

Supplement: Supplementary Figure 1 — Representative pictures for DI Chlorosis, Sporulation and Necrosis. For each of the three scored traits, representative pictures are given of the spectrum of the DI classes from 1 (susceptible) to 9 (resistant). [file Image_1.png]

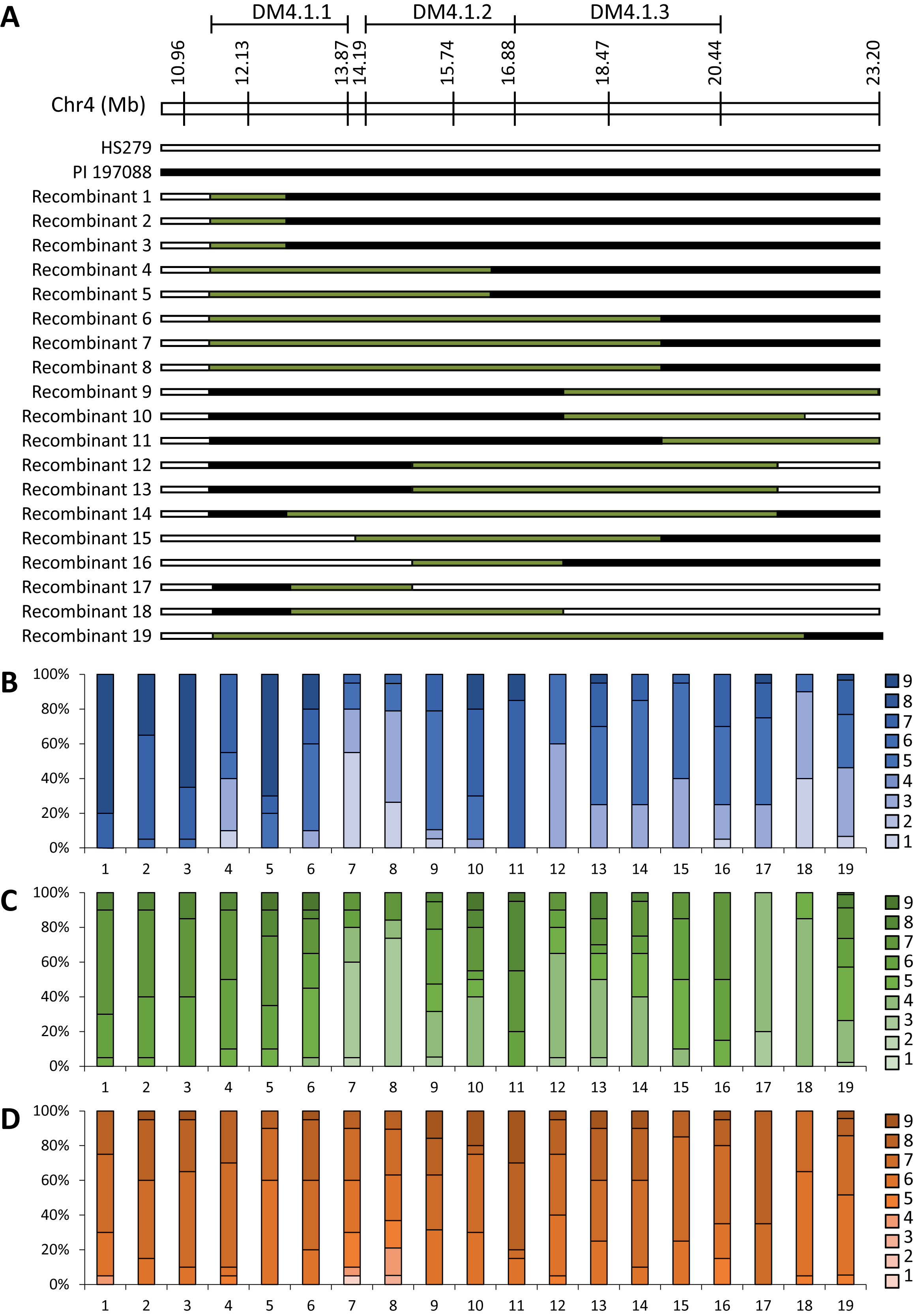

Supplement: Supplementary Figure 2 — Genotypic and phenotypic data of 19 recombinant families derived from a QTL isogenic introgression line. 19 F3BC3S3 individuals with recombination events within the DM4.1 locus were selected and genotyped, and progeny of these 19 plants were evaluated in a disease assay. (A) Bars represent the allele of genotypes at marker locations on the DM4.1 interval. Black bars indicate the PI 197088 allele, white bars indicate the HS279 allele, green bar represents heterozygosity. Populations were developed by self-fertilization of the 19 recombinants described in (A). 20 to 91 seedlings of each of the 19 populations were shown and used for a disease assay. Sporulation (B), chlorosis (C) and necrosis (D) were scored at 14 dpi on a scale from 1-9 as described before. Stacked bars represent the distribution of disease phenotypes in each of the 19 populations. [file Image_2.png]

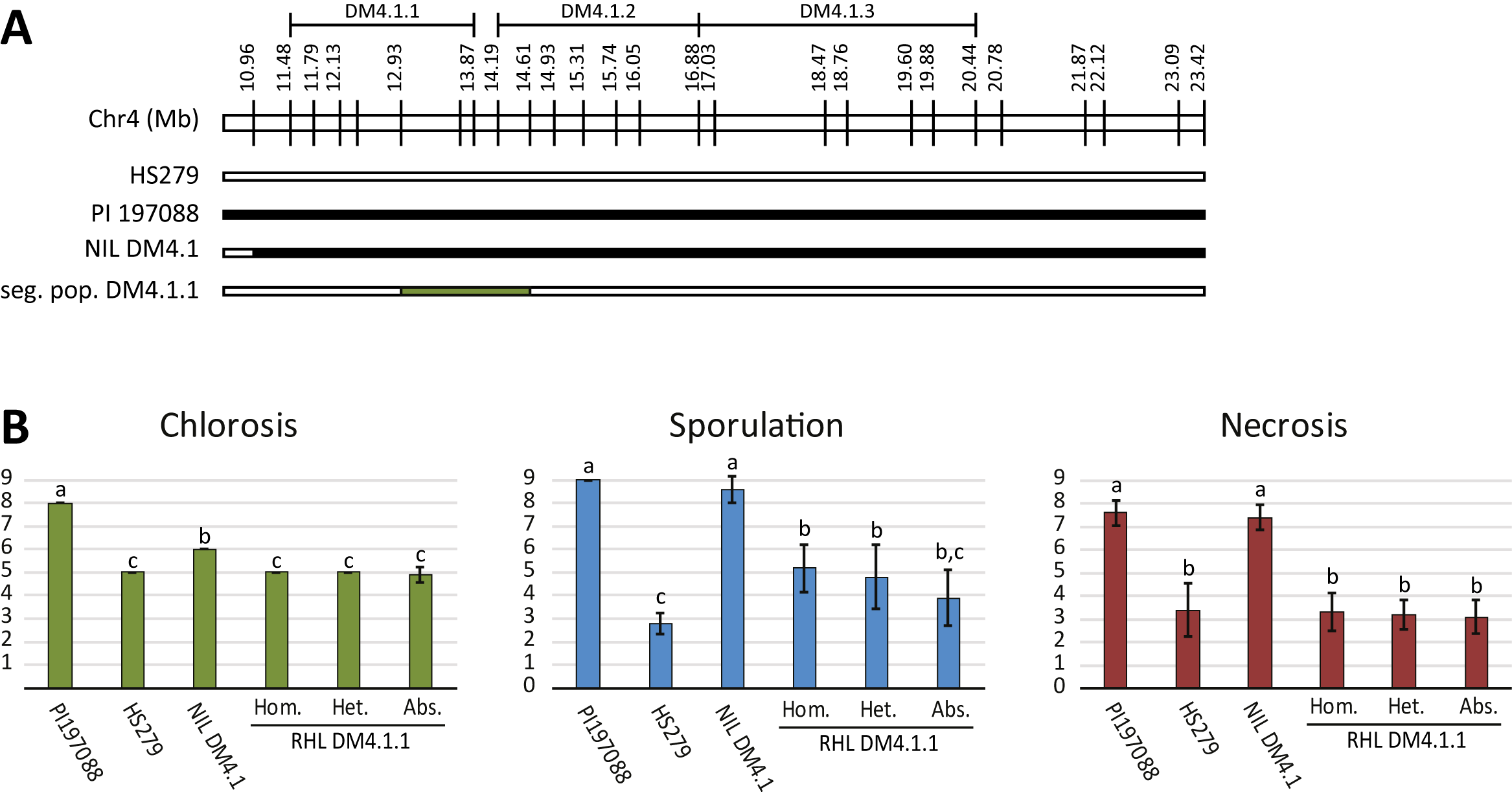

Supplement: Supplementary Figure 3 — P. cubensis disease test on RHL DM4.1.1. Similarly as described for Figure 3, a family was developed segregating for a partial introgression corresponding to part of subQTL DM4.1.1. (A) Bars represent the allele of genotypes at marker locations on the DM4.1 interval. Black bars indicate the PI 197088 allele, white bars indicate the HS279 allele, green bar represents heterozygosity. (B) The RHL was inoculated with P. cubensis, chlorosis was scored at 7 dpi whereas sporulation and necrosis were scored at 12 dpi. Bars represent average phenotype scores on a 1-9 scale ranging from susceptible to resistant. Error bars indicate standard deviations. Bars with different letters indicate statistically significant differences (Kruskal-Wallis test, p < 0.05). [file Image_3.png]

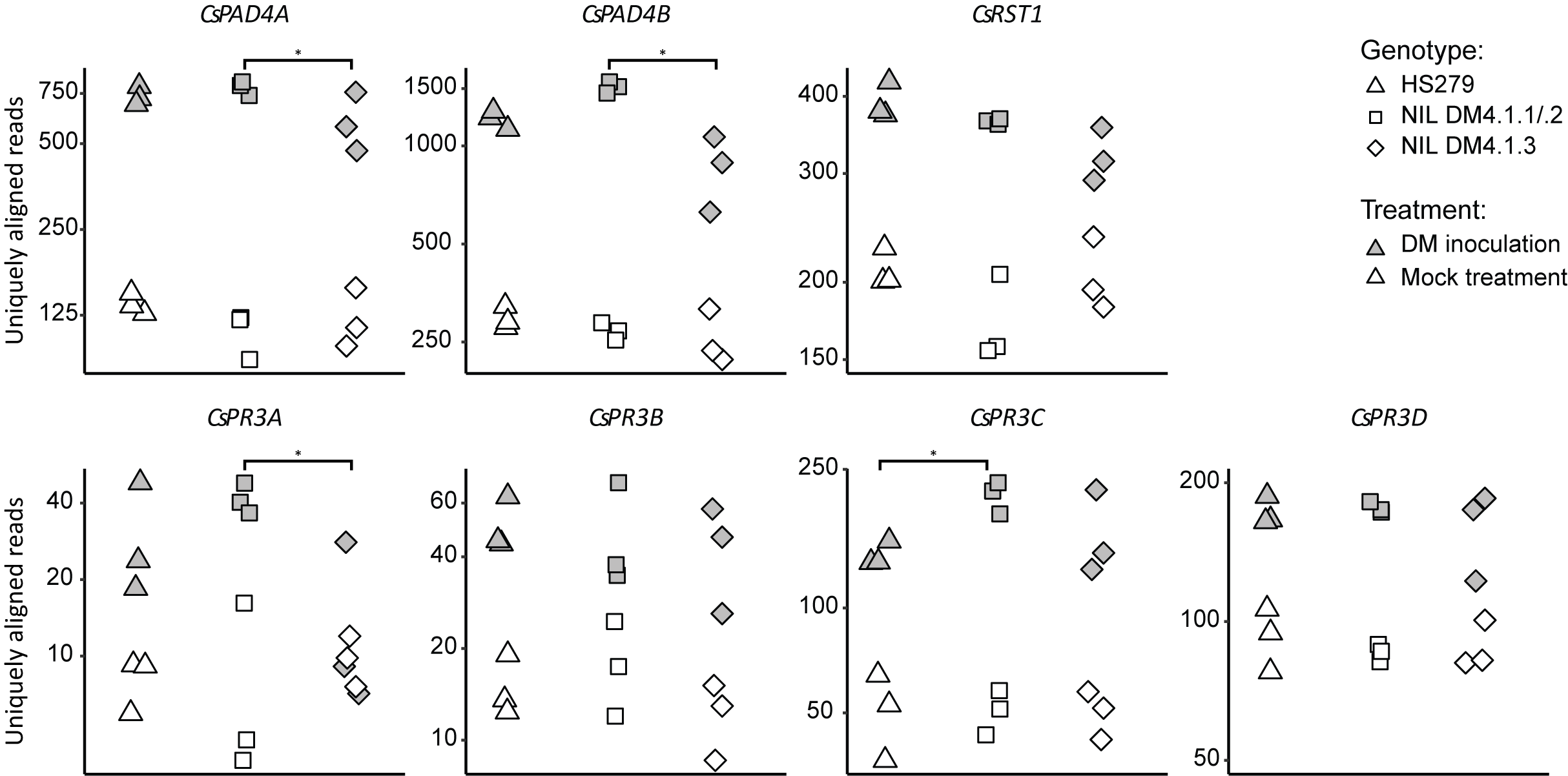

Supplement: Supplementary Figure 4 — Expression analysis of defense pathway genes. Expression data of cucumber homologs of known defense pathway genes were extracted from the RNAseq dataset, and plotted per sample on a logarithmic scale. Asterisks represent statistically significant differences between genotypes (adjusted p < 0.05). For all genes except CsPR3A, differences between treatments within genotypes were statistically significant (p < 0.05). [file Image_4.png]

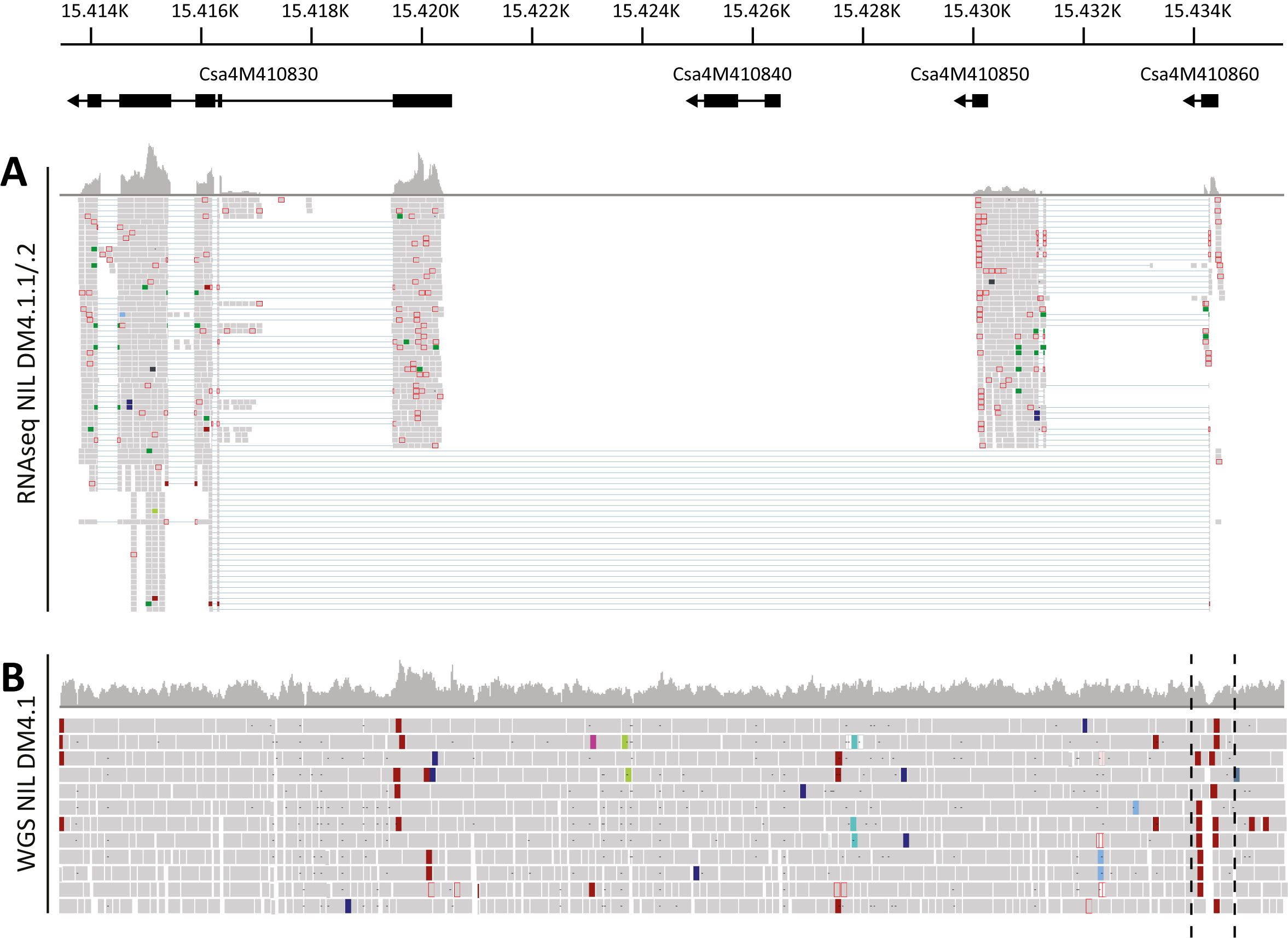

Supplement: Supplementary Figure 5 — RNAseq and WGS alignment RLK cluster. (A) RNAseq reads from P. cubensis inoculated NIL DM4.1.1/.2 aligning to the RLK cluster are visualized using the Integrative Genomics Viewer (IGV). Split reads are indicated with blue lines. A coverage graph is given above the aligned reads. (B) Whole genome sequencing reads of NIL DM4.1 aligning to the RLK cluster are visualized using the Integrative Genomics Viewer (IGV). A coverage graph is given above the aligned reads. Reads pairs with larger than expected or smaller than expected insert sizes are indicated in dark red and dark blue, respectively. Dotted lines denote the interval shown at greater resolution in Figure 6B, corresponding to predicted gene Csa4M410860. [file Image_5.png]

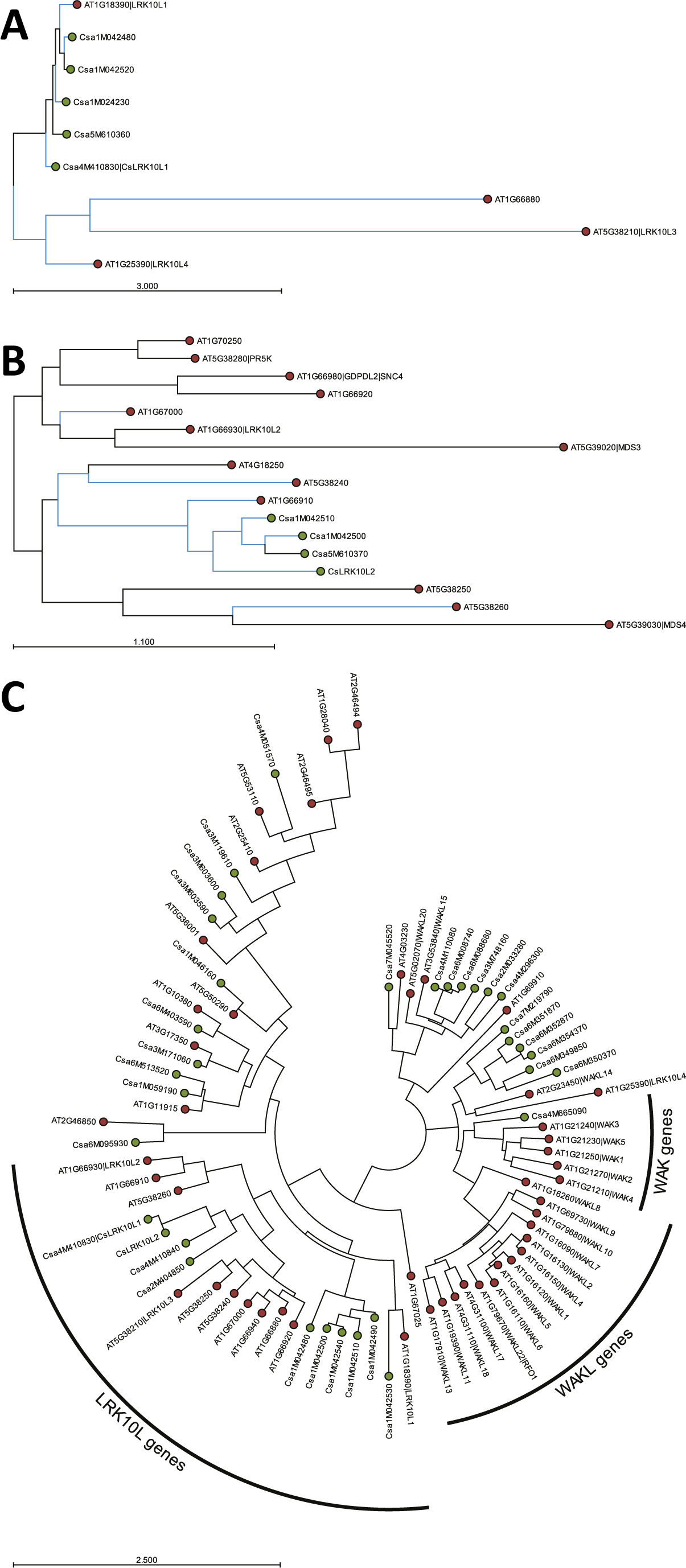

Supplement: Supplementary Figure 6 — Phylogenetic analysis CsLRK10L genes. Homologs of the kinase domains of CsLRK10L1 (A) and CsLRK10L2 (B) were identified using BLASTp against cucumber and Arabidopsis translated reference genomes, and used to construct phylogenetic (maximum likelihood) trees. Cucumber and Arabidopsis homologs are indicated with green and red circles, respectively. Branches leading to homologs with predicted oligogalacturonan (OG) binding and/or WAK-associated domains are colored blue. (C) A phylogenetic (maximum likelihood) tree was constructed based on predicted OG-binding domains of Arabidopsis and cucumber proteins. Clades containing the majority of previously annotated WAK, WAKL and LRK10L proteins are indicated. [file Image_6.png]

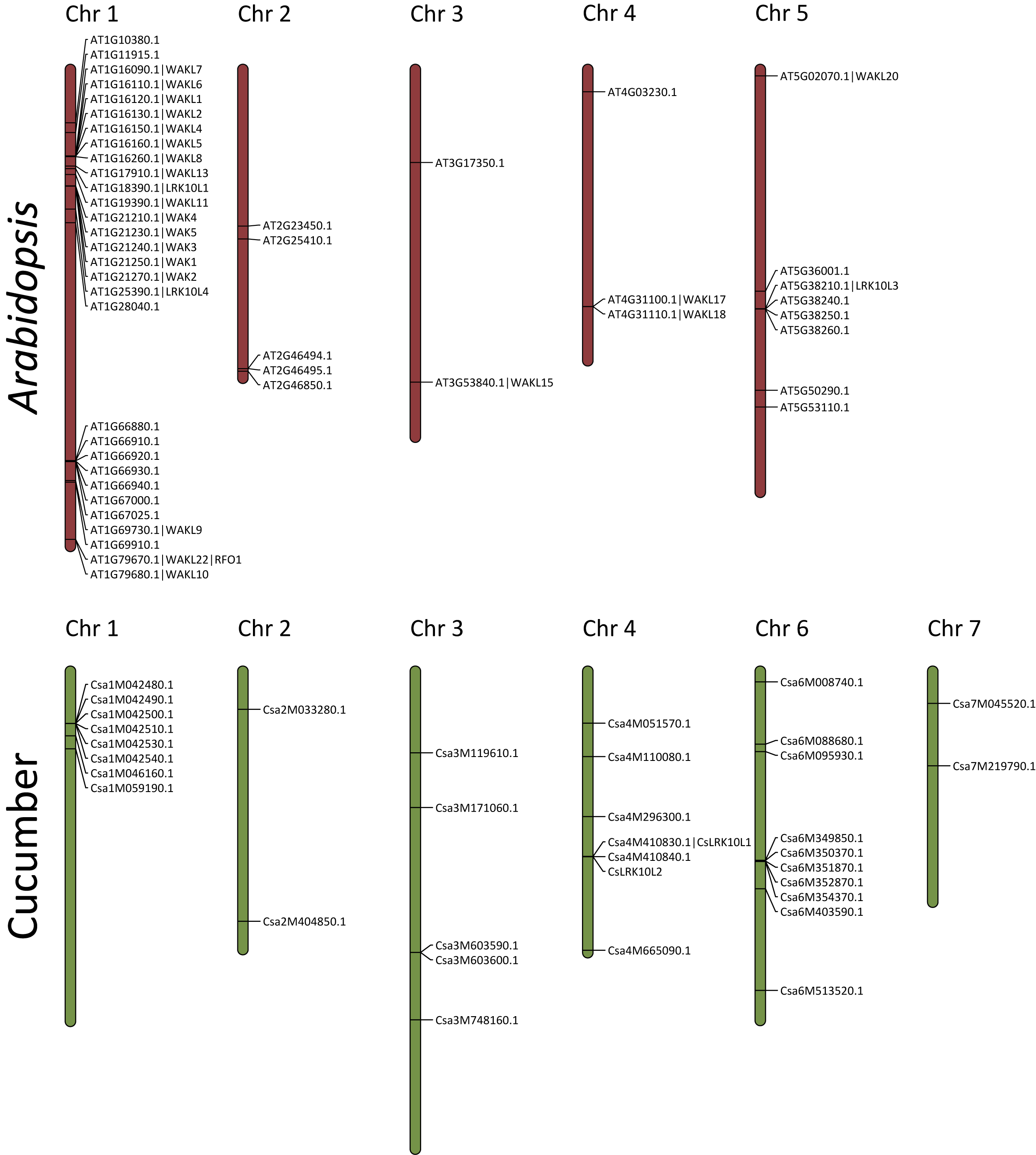

Supplement: Supplementary Figure 7 — Physical locations WAKL/LRK10L genes. Genomic positions of genes encoding proteins with predicted galacturonan-binding and/or WAK-associated domains were retrieved and visualized. [file Image_7.png]
